# Coronary microvascular function in patients with sepsis and myocardial injury: an invasive coronary physiology study

**DOI:** 10.1186/s13054-026-06179-3

**Published:** 2026-07-02

**Authors:** Samantha Lörstad, Per Åstrand, Patrik Gille-Johnson, Yunzhang Wang, Christina Ekenbäck, Fadi Jokhaji, Felix Böhm, Patrik Hjalmarsson, Shajan Shekarestan, Tomas Jernberg, Sara Tehrani, Kambiz Shahgaldi, Jonas Persson

**Affiliations:** 1 Department of Clinical Sciences, Division of Internal Medicine, Karolinska Institutet, Danderyd University Hospital, Stockholm, Sweden; 2 Internal Medicine Clinic, Danderyd University Hospital, Stockholm, Sweden; 3 Infectious Diseases Clinic, Danderyd University Hospital, Stockholm, Sweden; 4 Department of Clinical Sciences, Karolinska Institutet, Danderyd University Hospital, Stockholm, Sweden; 5 Department of Clinical Sciences, Division of Cardiovascular Medicine, Karolinska Institutet, Danderyd University Hospital, Stockholm, Sweden

**Keywords:** Sepsis, Coronary microcirculation, Myocardial injury, Troponin, Echocardiography, Coronary microvascular dysfunction.

## Abstract

**Background:**

Myocardial injury is common in sepsis and associated with increased mortality, but its underlying mechanisms remain incompletely understood. Coronary microvascular dysfunction (CMD) has been proposed as a contributor, although in vivo evidence is limited. We characterised coronary microvascular function in patients with sepsis and myocardial injury and its relationship with cardiac troponin release.

**Methods:**

Consecutive adults with sepsis (Sepsis-3 criteria) and myocardial injury (hs-cTnT ≥ 15 ng/L) were prospectively enrolled between June 2019 and December 2024. Patients underwent coronary angiography, invasive thermodilution-based assessment of coronary microvascular function, and transthoracic echocardiography after clinical stabilisation. CMD was defined as an index of microcirculatory resistance (IMR) > 25 and/or microvascular resistance reserve (MRR) ≤ 3. Associations between hs-cTnT concentrations and coronary microvascular indices, obstructive coronary artery disease (CAD), and echocardiographic variables were assessed using regression analyses. Coronary microvascular indices were compared with those of age-, sex-, and CAD-matched patients with chronic coronary syndrome (CCS) using mixed-effects models.

**Results:**

Fifty-five patients underwent coronary angiography and 49 completed invasive coronary microvascular assessment. Obstructive CAD was identified in 12/55 (22%). CMD was present in 30/49 (61%). Among these, 8/49 (16%) had elevated IMR only, 9/49 (18%) had functional CMD (MRR ≤ 3 and IMR ≤ 25), and 13/49 (27%) had structural CMD (MRR ≤ 3 and IMR > 25). Restricted cubic spline analyses demonstrated no evidence of associations between hs-cTnT and IMR (overall *P* = 0.899; non-linearity *P* = 0.687) or MRR (overall *P* = 0.987; non-linearity *P* = 0.954). CMD was associated with a higher prevalence of ventriculo-arterial uncoupling, right ventricular systolic dysfunction, and impaired right ventricular-pulmonary arterial coupling. Patients with sepsis demonstrated reduced coronary microvascular vasodilatory capacity compared with matched CCS controls (MRR 3.2 [IQR 2.4–4.5] vs. 4.0 [2.7–6.2]). IMR was similar between groups.

**Conclusions:**

CMD was common and heterogeneous and exhibited distinct phenotypes in patients with sepsis and myocardial injury. Measures of coronary microvascular function were not associated with troponin release. Previously unrecognised obstructive CAD was identified in 22% of patients, supporting consideration of underlying CAD in patients with sepsis and myocardial injury.

**Trial registration:**

ClinicalTrials.gov identifier NCT06294730.

**Graphical abstract:**

**Figure:**
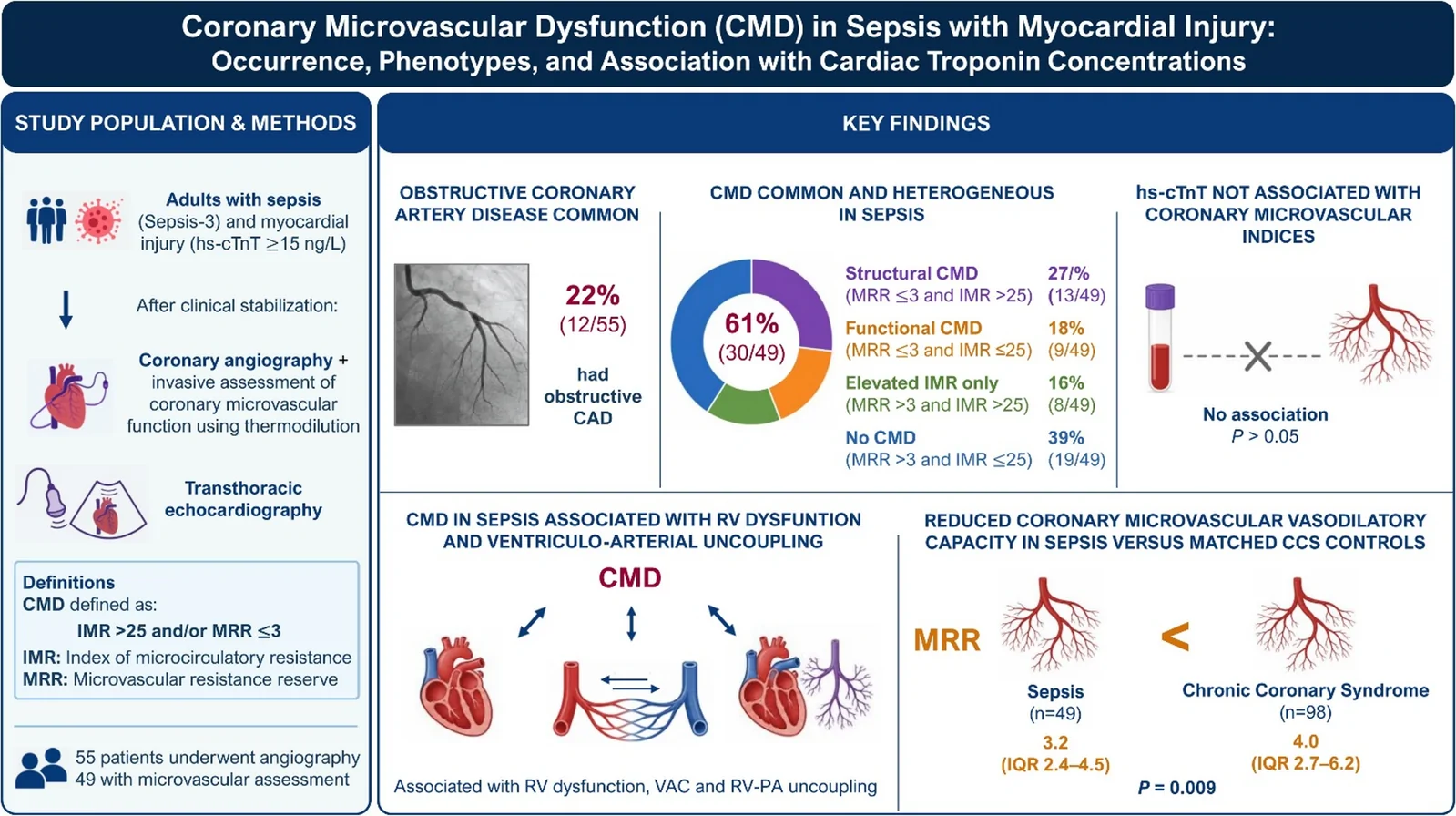

**Supplementary Information:**

The online version contains supplementary material available at https://doi.org/10.1186/s13054-026-06179-3.

## Background

Myocardial injury is common in sepsis and consistently associated with adverse outcomes, yet its underlying mechanisms remain incompletely understood [1, 2]. Coronary microvascular dysfunction (CMD) has been proposed as a contributing mechanism [3]. Histopathological studies demonstrate microvascular alterations alongside cardiomyocyte damage, supporting a mechanistic link between coronary microvascular injury and myocardial injury [4, 5]. However, CMD has not been systematically characterised in vivo in patients with sepsis.

The contribution of obstructive coronary artery disease (CAD) to myocardial injury in sepsis also remains uncertain. Existing angiographic studies have largely been restricted to highly selected patients with suspected acute coronary syndromes, and the frequency of obstructive CAD in patients with sepsis-associated myocardial injury remains poorly defined [6–8].

Sepsis is fundamentally a disorder of the microcirculation, characterised by widespread endothelial and glycocalyx injury, capillary leak, and impaired microvascular perfusion, leading to multi-organ dysfunction, often with cardiac involvement [9, 10]. These alterations may manifest within the coronary circulation as acute CMD through impaired vasoreactivity, microvascular obstruction, and interstitial oedema, potentially contributing to myocardial injury [10, 11].

In this study, we performed invasive coronary angiography and assessment of coronary microvascular function in patients with sepsis and myocardial injury, defined by elevated plasma high-sensitivity cardiac troponin T (hs-cTnT) concentrations. We hypothesised that greater impairment in microvascular function would be associated with higher hs-cTnT concentrations. In addition, we characterised the frequency and physiological phenotypes of CMD in sepsis and evaluated the occurrence of underlying obstructive epicardial CAD. We also explored associations between coronary physiological indices, echocardiographic findings, and cardiac biomarker concentrations.

Coronary microvascular function was quantified using established invasive thermodilution-derived indices, including the index of microcirculatory resistance (IMR) and microvascular resistance reserve (MRR) [12, 13]. We also compared microvascular findings with those of a matched cohort of patients with chronic coronary syndrome (CCS) to provide physiological context for the magnitude and patterns of CMD observed in sepsis.

## Methods

### Study design and objectives

This prospective observational study aimed to characterise coronary microvascular function in patients with sepsis-associated myocardial injury using invasive thermodilution-based assessment. The primary objective was to examine the association between plasma hs-cTnT concentrations and IMR, an invasive index of hyperaemic coronary microvascular resistance.

Pre-specified secondary objectives were to evaluate the association between plasma hs-cTnT concentrations and obstructive coronary artery disease (CAD), additional thermodilution-derived coronary microvascular indices, and echocardiographic measures of cardiac structure and function. Additional secondary objectives were to characterise the occurrence and physiological phenotypes of CMD.

Exploratory objectives included assessing associations of plasma NT-proBNP concentrations with coronary microvascular indices and echocardiographic measures of cardiac structure and function. Coronary microvascular function in patients with sepsis was also compared with that of a matched control cohort with CCS.

### Patient population

In the *COronary Microcirculation and Troponin Elevation in Septic Shock* (COMTESS; ClinicalTrials.gov identifier: NCT06294730; registered March 2, 2024), we prospectively enrolled consecutive adults admitted to the intensive or intermediate care unit at Danderyd University Hospital. All patients with sepsis admitted to these units were prospectively screened for eligibility.

Eligible patients were aged 40–85 years, met the Sepsis-3 diagnostic criteria for sepsis or septic shock, had an expected survival >1 year, and demonstrated myocardial injury, defined as a plasma hs-cTnT concentration ≥ 15 ng/L within 48 h of sepsis onset [14, 15]. Patients were screened on the basis of hs-cTnT elevation irrespective of symptoms suggestive of acute coronary syndrome or prior suspicion of CAD.

Exclusion criteria included prior coronary artery bypass grafting, pre-admission left ventricular (LV) ejection fraction ≤ 39%, chronic kidney disease with a pre-admission estimated glomerular filtration rate (eGFR) < 30 mL/min/1.73 m², and infective endocarditis. Patients with persistent clinical instability beyond day 8 (ongoing circulatory shock requiring vasopressors or respiratory failure requiring ventilatory support) were excluded. Patients were also excluded when invasive coronary angiography was considered unsafe or unfeasible (full criteria are provided in the Supplementary Methods). Detailed patient screening, enrolment, and reasons for exclusion are shown in Supplementary Figure S1.

Patients underwent coronary angiography with invasive assessment of coronary microvascular function and transthoracic echocardiography (TTE) following clinical stabilisation, between days 2 and 10 after sepsis onset. Invasive assessment was performed after haemodynamic stabilisation to minimise the influence of shock and vasoactive therapy on coronary physiological measurements and to ensure procedural safety.

The study was approved by the Swedish Ethical Review Authority, conducted in accordance with the Declaration of Helsinki, and all participants provided written informed consent.

### Coronary angiography and coronary microvascular assessment

Coronary angiography was performed according to standard clinical protocols. Obstructive CAD was defined as ≥ 50% diameter stenosis or fractional flow reserve (FFR) ≤ 0.80 in an epicardial coronary artery ≥ 2.5 mm in diameter [16].

Coronary flow indices were obtained by thermodilution [12, 13, 17]. Following administration of ≥ 100 µg intracoronary nitroglycerine, a thermistor-equipped pressure wire (PressureWire™ X, Abbott Vascular Inc., CA, USA) was advanced > 70 mm into the left anterior descending artery (LAD) and connected to dedicated acquisition software (Coroventis Research AB, Uppsala, Sweden).

At rest, three intracoronary bolus injections of saline (3 mL each) were administered to determine mean transit time. Aortic and distal coronary pressures were simultaneously recorded. Maximal hyperaemia was induced using intravenous adenosine (167 µg/kg/min), followed by repeat triplicate saline injections to obtain hyperaemic mean transit time. Hyperaemic aortic and distal coronary pressures were simultaneously recorded [12, 17].

Resting and hyperaemic coronary blood flow (CBF) were defined as the inverse of mean transit time [18]. Baseline resistance index (BRI) and index of microcirculatory resistance (IMR) were calculated as measures of resting and hyperaemic microvascular resistance, respectively. Collateral-corrected IMR was used for all analyses to account for flow-limiting CAD [19, 20]. Microvascular resistance reserve (MRR) was calculated as a pressure-corrected index of coronary microvascular vasodilatory capacity. Unlike coronary flow reserve (CFR), which is influenced by both epicardial and microvascular disease, MRR is a specific index of microvascular vasodilator capacity [13, 21]. Detailed definitions and calculation formulas are provided in Supplementary Table S1.

CMD was defined as MRR ≤ 3 and/or IMR > 25 according to established invasive coronary physiology thresholds [13, 16]. Patients were further classified into four physiological phenotypes: no CMD (MRR > 3 and IMR ≤ 25); elevated IMR only (MRR > 3 and IMR > 25); functional CMD (MRR ≤ 3 and IMR ≤ 25); and structural CMD (MRR ≤ 3 and IMR > 25) [11, 13, 16, 22].

### Echocardiography

TTE was performed using Vivid E9 or Vivid E95 ultrasound systems (GE Healthcare) equipped with an M5Sc transducer and analysed according to established guidelines [23]. Comprehensive echocardiographic assessment of cardiac structure and function was performed by certified sonographers or clinical physiologists and reviewed by a single certified assessor blinded to clinical data.

Detailed echocardiographic acquisition, measurements, thresholds, and classification criteria for diastolic dysfunction are provided in the Supplementary Methods.

### Cardiac biomarkers

Plasma hs-cTnT (Roche Diagnostics, Elecsys Assay 2) and N-terminal pro–B-type natriuretic peptide (NT-proBNP; Roche Diagnostics, Elecsys Assay 3) were measured serially during the first 48 h after sepsis onset. Sampling was intended at approximately 12, 24, and 48 h according to the study protocol. In practice, timing was guided by local routine and clinical indication, and additional samples were often obtained at shorter intervals (1–3 h) to assess dynamic changes. hs-cTnT measurements were repeated until concentrations stabilised or declined, and the highest value recorded during the first 48 h was used to estimate early myocardial injury burden.

### Clinical data collection

Baseline demographics, comorbidities identified from International Classification of Diseases, Tenth Revision (ICD-10) codes recorded prior to admission, in-hospital laboratory results and treatments, primary site of infection, microbiological findings, and vital status were prospectively collected from the electronic health record. Sepsis onset was defined as the earliest documented time at which sepsis-related physiological abnormalities included in the National Early Warning Score 2 were recorded in the electronic health record. A 12-lead electrocardiogram was obtained within four hours of sepsis onset. Atrial fibrillation was confirmed by findings on electrocardiography or continuous telemetry monitoring.

### Control population

Controls matched for age, sex and number of obstructive CAD lesions were selected from a cohort of 506 patients with CCS who underwent clinically indicated elective coronary angiography and thermodilution-derived assessment of coronary microvascular function at our institution [24]. Controls were matched 2:1 to the 49 sepsis cases with complete coronary microvascular measurements in the LAD. The CCS cohort was selected as a reference population to relate the magnitude and patterns of CMD phenotypes in sepsis to CCS-patients. CMD is a recognised cause of myocardial ischaemia and symptoms in chronic coronary syndrome, and invasive physiological assessment has an established role in its evaluation and phenotypic classification [16].

### Statistical analysis

Sample size estimation was based on detecting a correlation coefficient of *r* = 0.40 between hs-cTnT and IMR with 80% power and a two-sided α of 0.05, yielding a required sample size of 47 patients with complete coronary microvascular measurements.

Continuous variables are presented as mean (standard deviation) or median (interquartile range), and categorical variables as counts (percentages). Between-group comparisons were performed using Student’s t-test or Mann-Whitney U test for continuous variables and the χ² test or Fisher’s exact test for categorical variables, as appropriate.

Plasma hs-cTnT concentrations and coronary microvascular and echocardiographic variables with right-skewed distributions were natural logarithm-transformed prior to regression analyses.

Associations between ln(hs-cTnT) and ln(NT-proBNP) concentrations and coronary physiological and echocardiographic variables were assessed using univariable linear regression. Restricted cubic spline models were used to assess potential non-linear relationships.

Comparisons of coronary microvascular indices between patients with sepsis and matched CCS controls were performed using linear mixed-effects models.

A two-sided p-value < 0.05 was considered statistically significant. Statistical analyses were performed using IBM SPSS Statistics version 30.0.0.0 and R version 4.4.1.

## Results

### Study population and procedural timing

**Fig. 1:**
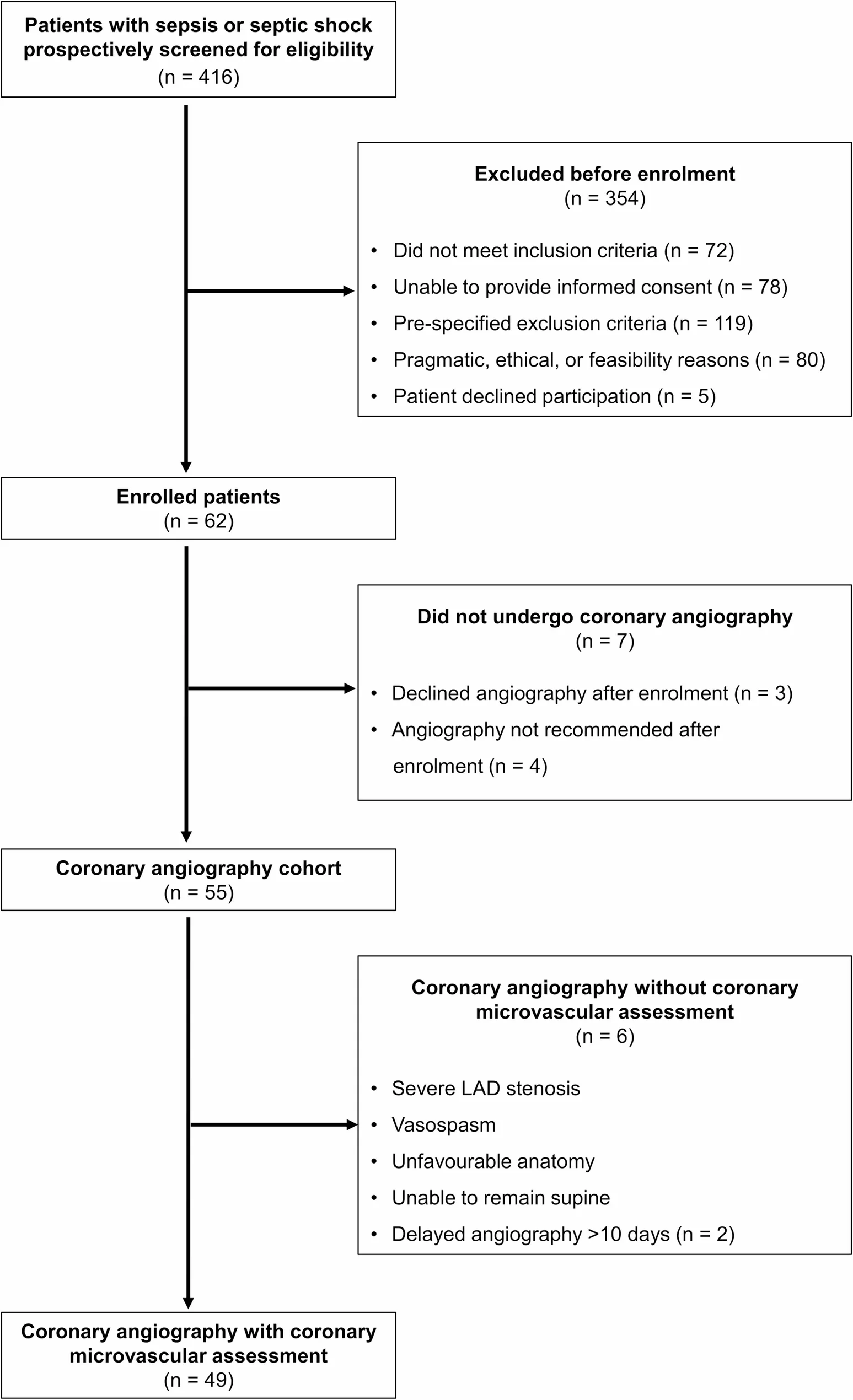
Patient screening, enrolment, and study flow

Between June 2019 and December 2024, 416 consecutive patients with sepsis or septic shock were screened for eligibility (Fig. 1). Of the 62 enrolled patients, 55 (89%) underwent coronary angiography and comprised the study population. Coronary microvascular physiological assessment was performed in 49 of 55 patients (89%); in the remaining six patients, microvascular assessment was not performed for anatomical, technical, or timing-related reasons (Fig. 1).

Serial hs-cTnT measurements were available in 53 of 55 patients (96%). TTE was performed 3.0 ± 1.8 days after sepsis onset and coronary angiography 4.2 ± 1.9 days after sepsis onset, with a mean interval of 1.1 ± 1.9 days between the two modalities.

### Baseline characteristics and in-hospital course

The cohort (*n* = 55) was elderly, predominantly male, and had low baseline frailty (Table 1). Patients with both sepsis and septic shock were represented. Genitourinary and respiratory infections were the most common infection sources, and among patients with positive blood cultures, gram-negative rods predominated.

**Table 1:** Baseline characteristics and hospital course according to the presence of obstructive coronary artery disease on coronary angiography

Variable	Total (*n* = 55)	No obstructive CAD (*n* = 43)	Obstructive CAD (*n* = 12)
**Demographics**			
Age, years, median (IQR)	75 (67–79)	73 (64–78)	76 (75–80)
Female sex, n (%)	16 (29)	15 (35)	1 (8)
Current or former smoker, n (%)	34 (62)	25 (58)	9 (75)
BMI, kg/m^2^, median (IQR)	25 (24–29)	26 (24–29)	25 (24–28)
Clinical Frailty Scale, median (IQR)	3 (2–4)	3 (2–4)	3 (2–6)
**Comorbid history**			
Previous myocardial infarction, n (%)	4 (7)	3 (7)	1 (8)
History of atrial fibrillation, n (%)	12 (22)	9 (21)	3 (25)
History of heart failure, n (%)	6 (11)	5 (12)	1 (8)
Previous stroke/TIA, n (%)	4 (7)	3 (7)	1 (8)
Hypertension, n (%)	37 (67)	26 (61)	11 (92)
Diabetes mellitus, n (%)	16 (29)	10 (23)	6 (50)
Pre-admission eGFR, mL/min/1.73 m^2^, median (IQR)	69 (55–79)	72 (56–81)	58 (51–71)
Cancer (previous/current), n (%)	18 (33)	13 (30)	5 (42)
**Hospital course**			
Septic shock (Sepsis-3), n (%)	34 (62)	25 (58)	9 (75)
ICU/IMCU length of stay, days, median (IQR)	3 (2–4)	4 (2–4)	3 (2–4)
Arterial pO_2_ at admission, kPa, mean ± SD	8.5 ± 2.1	8.5 ± 2.3	8.2 ± 1.2
Normal electrocardiogram on admission, n (%)	17 (31)	13 (30)	4 (33)
Fluids before norepinephrine, L, median (IQR)	3 (2–4)	3 (2–4)	3 (2–3)
Norepinephrine treatment, n (%)	35 (64)	26 (61)	9 (75)
Norepinephrine duration, h, median (IQR)	33 (19–54)	42 (20–63)	26 (14–37)
Norepinephrine peak dose (µg/kg/min), median (IQR)	0.12 (0.08–0.25)	0.12 (0.08–0.40)	0.10 (0.05–0.18)
Additional vasopressor or inotrope, n (%)	7 (13)	7 (16)	0 (0)
Highest recorded heart rate, bpm, mean ± SD	111 ± 25	110 ± 26	118 ± 20
Peak SOFA score, median (IQR)	7 (5–9)	7 (5–9)	8 (6–9)
Lactate, mmol/L, median (IQR)	4.0 (2.8–5.3)	3.9 (2.8–5.1)	5.6 (3.1–7.6)
hs-cTnT (ng/L), median (IQR)	110 (63–229)	106 (55–227)	202 (73–407)
NT-proBNP, ng/L, median (IQR)	6890 (2620–13 000)	5200 (2155–11 000)	9690 (5170–13 900)
Acute kidney injury, n (%)	37 (67)	30 (70)	7 (58)
CRP, mg/L, mean ± SD	242 ± 108	239 ± 107	256 ± 117
Haemoglobin g/L, mean ± SD	122 ± 16	122 ± 16	121 ± 15
Haematocrit, %, mean ± SD	34 ± 5	34 ± 5	33 ± 5
Platelet count,$\:\times\:$10^9^/L, median (IQR)	151 (118–203)	141 (114–207)	173 (123–197)
New-onset atrial fibrillation, n (%)	10 (18)	8 (19)	2 (17)
**Infection**			
Respiratory, n (%) Gastrointestinal, n (%) Genitourinary, n (%) Skin/soft tissue, n (%)	18 (33) 5 (9) 28 (51) 4 (7)	13 (30) 3 (7) 24 (56) 3 (7)	5 (42) 2 (17) 4 (33) 1 (8)
Positive blood culture, n (%) Gram-positive cocci, n (%) Gram-negative rods, n (%) Gram-positive anaerobes, n (%) Gram-negative cocci, n (%)	42 (76) 10 (18) 29 (53) 1 (2) 1 (2)	32 (74) 7 (16) 24 (55) 1 (2) 0 (0)	10 (77) 3 (25) 5 (42) 0 (0) 1 (8)

Data are presented as median (interquartile range), mean ± standard deviation, or number (%). Acute kidney injury was defined according to the Kidney Disease: Improving Global Outcomes (KDIGO) criteria as any of the following: an increase in serum creatinine ≥ 26.5 µmol/L (≥ 0.3 mg/dL) within 48 h, or an increase in serum creatinine to ≥ 1.5 times baseline known or presumed to have occurred within the 7 days prior to sepsis onset. All laboratory values represent the most abnormal measurement within 48 h of sepsis onset. BMI = body mass index; CAD = coronary artery disease; CRP = C-reactive protein; eGFR = estimated glomerular filtration rate; hs-cTnT = high-sensitivity cardiac troponin T; ICU = intensive care unit; IMCU = intermediate care unit; NT-proBNP = N-terminal pro–B-type natriuretic peptide; SOFA = sequential organ failure assessment; TIA = transient ischemic attack

Patients with obstructive CAD were older, more often male, and more frequently had hypertension, diabetes mellitus, smoking history, and reduced pre-admission renal function than those without obstructive CAD. They also presented with higher lactate, hs-cTnT, and NT-proBNP concentrations and more frequently fulfilled criteria for septic shock. Infection source and microbiological findings were broadly similar between groups, as were most other baseline and in-hospital clinical characteristics.

### Coronary angiographic findings

Among the 55 patients undergoing coronary angiography, obstructive CAD was identified in 12 (22%). Only one had a documented history of previous myocardial infarction. Most patients without obstructive CAD had angiographically normal coronary arteries or only mild non-obstructive atherosclerotic disease.

Coronary revascularisation with percutaneous coronary intervention (PCI) was performed in 6 of the 12 patients with obstructive CAD during the index hospitalisation. Patients who did not undergo revascularisation were managed conservatively because of chronic total occlusions, small-vessel disease, lesion complexity, or patient preference. One additional patient initially classified as having non-obstructive CAD with extensive diffuse atherosclerosis underwent repeat coronary angiography with PCI two months after sepsis onset.

### Coronary microvascular function

Among the 49 patients undergoing complete coronary physiological assessment, CMD, defined as IMR > 25 and/or MRR ≤ 3, was present in 30 patients (61%). Patients with CMD were of similar age and sex distribution to those without CMD. However, they had lower habitual eGFR (66 [IQR 56–74] vs. 78 [68–86] mL/min/1.73 m²; *P* = 0.025) and higher peak C-reactive protein (CRP) concentrations (260 ± 106 vs. 196 ± 96 mg/L; *P* = 0.041). Median NT-proBNP concentrations also tended to be higher in patients with CMD (9910 [IQR 2620–16 900] vs. 4000 [1260–8823] ng/L; *P* = 0.071), whereas hs-cTnT concentrations, illness severity, vasopressor requirements, and infection characteristics were otherwise broadly similar between groups.

**Fig. 2:**
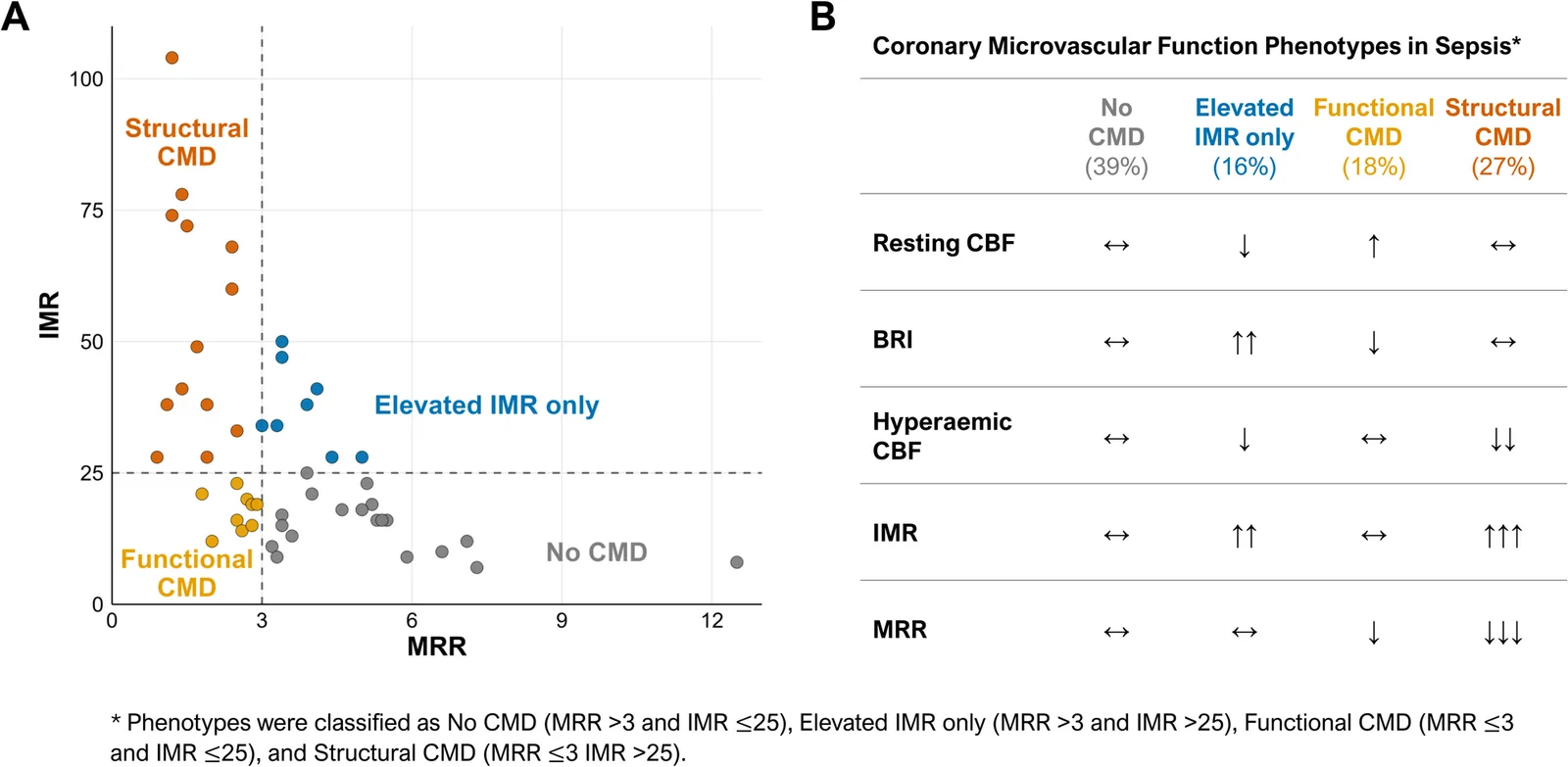
Coronary microvascular dysfunction phenotypes in sepsis. (**A**) Distribution of individual patients according to the index of microcirculatory resistance (IMR) and microvascular resistance reserve (MRR). Dashed lines indicate the predefined thresholds for abnormal MRR (≤ 3) and IMR (> 25), defining four physiological phenotypes. Coronary microvascular dysfunction (CMD) was defined as MRR ≤ 3 and/or IMR > 25. (**B**) Schematic summary of coronary physiological profiles observed across the four coronary microvascular function phenotypes. Arrow direction indicates the direction of change relative to patients with no CMD. Increasing numbers of arrows indicate greater differences. Phenotype frequencies are shown in parentheses. Quantitative data are presented in Table 2. BRI = baseline resistance index; CBF = coronary blood flow

Coronary microvascular function was heterogeneous: 19 patients (39%) had no CMD, 8 (16%) had elevated IMR only, 9 (18%) had functional CMD, and 13 (27%) had structural CMD (Fig. 2A).

**Table 2:** Coronary microvascular function across physiological phenotypes

Coronary microvascular indices	Total (*n* = 49)	No CMD (*n* = 19)	Elevated IMR only (*n* = 8)	Functional CMD (*n* = 9)	Structural CMD (*n* = 13)
Resting CBF, median (IQR)	1.1 (0.7–1.6)	1.2 (0.8–1.5)	0.6 (0.5–0.7)	1.7 (1.5–2.4)	1.0 (0.8–1.5)
BRI, median (IQR)	78 (48–105)	78 (47–86)	136 (110–161)	38 (35–52)	82 (51–113)
Hyperaemic CBF, median (IQR)	3.2 (1.8–4.3)	4.3 (3.4–6.4)	1.8 (1.7–2.1)	3.4 (3.0–4.2)	1.4 (1.2–2.4)
IMR, median (IQR)	21 (15–38)	16 (10–18)	35 (29–45)	19 (14–20)	49 (35–73)
MRR, median (IQR)	3.3 (2.2–4.8)	5.1 (3.6–5.9)	3.7 (3.4–4.3)	2.6 (2.3–2.8)	1.5 (1.2–2.1)
FFR, mean ± SD	0.90 ± 0.07	0.89 ± 0.07	0.91 ± 0.09	0.88 ± 0.07	0.92 ± 0.07

Coronary microvascular physiological assessment was available in 49/55 patients undergoing coronary angiography. Coronary microvascular phenotypes were classified as No CMD (MRR > 3 and IMR ≤ 25), Elevated IMR only (MRR > 3 and IMR > 25), Functional CMD (MRR ≤ 3 and IMR ≤ 25), and Structural CMD (MRR ≤ 3 and IMR > 25). BRI = baseline resistance index; CBF = coronary blood flow; CMD = coronary microvascular dysfunction; FFR = fractional flow reserve; IMR = index of microcirculatory resistance; MRR = microvascular resistance reserve

Distinct physiological patterns were observed across the coronary microvascular phenotypes. Patients with elevated IMR only demonstrated preserved vasodilatory capacity (MRR > 3) despite lower resting and hyperaemic CBF and higher resting (BRI) and hyperaemic microvascular resistance (IMR) than patients with no CMD (Table 2; Fig. 2B). Patients with functional CMD exhibited higher resting CBF and lower resting microvascular resistance (BRI). Patients with structural CMD had the highest IMR values together with the lowest hyperaemic CBF and MRR values, reflecting both increased hyperaemic microvascular resistance and impaired vasodilatory capacity.

Several coronary physiological indices exhibited numerical differences according to obstructive CAD status but none reached statistical significance (Supplementary Table S2).

### Cardiac biomarkers in relation to coronary microvascular function and echocardiography parameters

Plasma concentrations of hs-cTnT and NT-proBNP were markedly elevated and demonstrated right-skewed distributions (Supplementary Figure S2). hs-cTnT concentrations ranged from 19 to 3360 ng/L and NT-proBNP concentrations from 111 to > 35,000 ng/L.

**Fig. 3:**
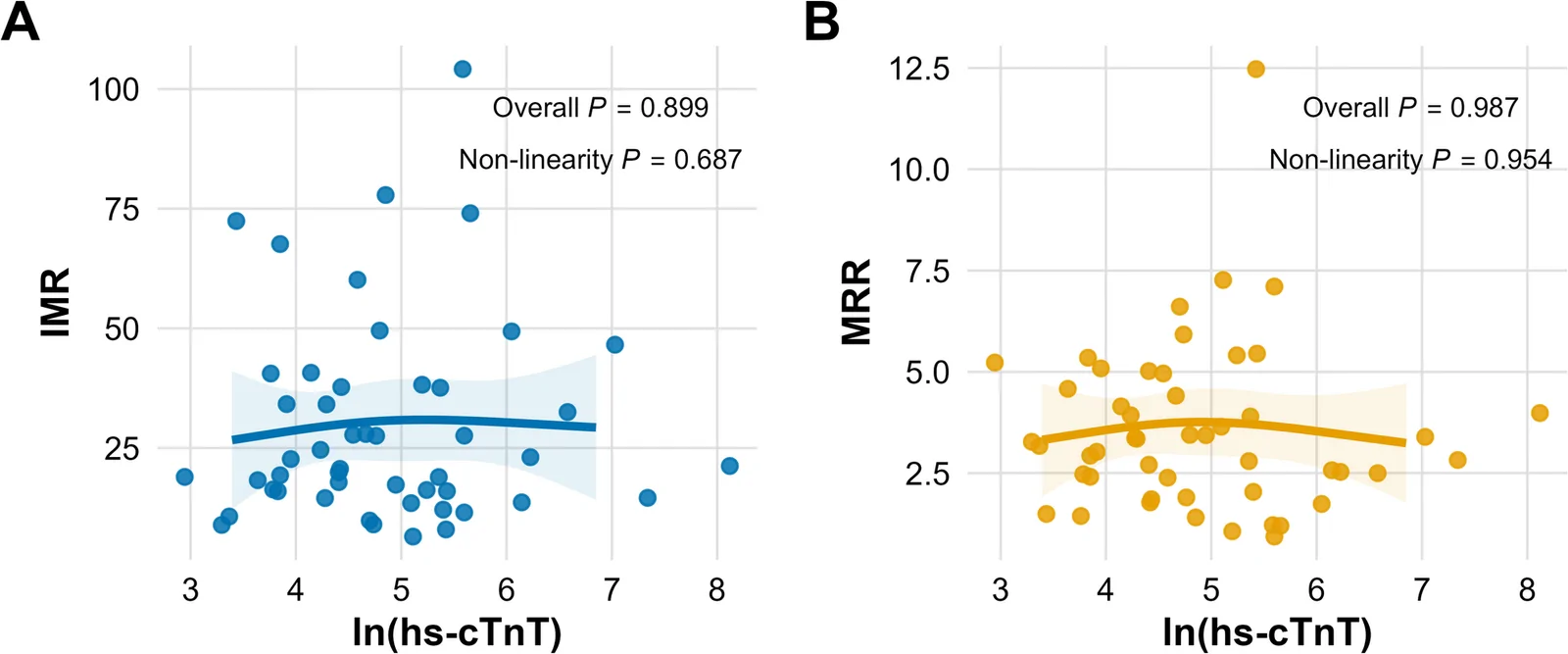
Restricted cubic spline analyses of the associations between ln-transformed high-sensitivity cardiac troponin T (hs-cTnT) concentrations and (**A**) index of microcirculatory resistance (IMR) and (**B**) microvascular resistance reserve (MRR) in patients undergoing invasive coronary microvascular assessment (*n* = 49). Solid lines represent fitted spline curves, and shaded areas represent 95% confidence intervals

Neither IMR nor MRR, analysed as continuous physiological measures, was associated with hs-cTnT concentrations (Fig. 3). NT-proBNP concentrations were likewise not associated with IMR (overall *P* = 0.504; non-linearity *P* = 0.774) or MRR (*P* = 0.878; non-linearity *P* = 0.895).

hs-cTnT concentrations were associated with a consistent pattern of left ventricular systolic dysfunction, including lower left ventricular ejection fraction (LVEF), lower cardiac output, reduced left ventricular longitudinal wall fractional shortening, and regional wall motion abnormalities (Supplementary Figure S3). NT-proBNP concentrations were associated with a broader pattern of cardiac and haemodynamic dysfunction involving left and right ventricular function, diastolic function, pulmonary pressures, and ventriculo-arterial coupling (Supplementary Figure S3).

### Echocardiographic findings in relation to coronary microvascular dysfunction

Among the 55 patients undergoing coronary angiography, left ventricular ejection fraction (LVEF) was generally preserved (median 55% [IQR 47–60]), whereas ventriculo-arterial uncoupling, diastolic dysfunction, and right ventricular abnormalities were common (Supplementary Table S3).

CMD was associated with a higher prevalence of ventriculo-arterial uncoupling, right ventricular systolic dysfunction, and impaired right ventricular-pulmonary arterial coupling, reflected by lower tricuspid annular plane systolic excursion (TAPSE), lower right ventricular fractional area change (RV-FAC), and lower TAPSE/systolic pulmonary artery pressure (TAPSE/SPAP) ratios (Table 3).

**Table 3:** Echocardiographic characteristics according to the presence of coronary microvascular dysfunction (*n* = 49)

Variable	No CMD (*n* = 19)	CMD (*n* = 30)	*P*
**Left ventricular systolic function**			
Cardiac output, L/min, mean ± SD	5.8 ± 1.3	5.6 ± 1.5	0.460
LVEF, %, median (IQR)	57 (45–62)	51 (45–59)	0.417
Regional wall motion abnormality, n (%)	7 (37)	7 (24)	0.344
LV-LWFS, %, median (IQR)	12 (10–14)	12 (10–14)	0.591
**Ventriculo-arterial coupling**			
VAC > 1.0, n (%)	9 (47)	23 (78)	0.036
**Left ventricular diastolic function**			
E/e´ ratio, median (IQR) (*n* = 47)	10 (8–12)	10 (8–12)	0.860
Diastolic function classification, n (%) (*n* = 48) - Normal diastolic function - Impaired relaxation with normal filling pressures - Impaired relaxation with elevated filling pressures	10 (53) 4 (21) 5 (26)	8 (28) 6 (21) 15 (52)	0.157
**Right ventricular systolic function**			
TAPSE, mm, mean ± SD	21 ± 5	18 ± 4	0.008
RV-FAC, %, mean ± SD	38 ± 8	32 ± 7	0.012
RV-FAC < 35%, n (%)	5 (29)	18 (69)	0.010
**Right ventricular-pulmonary vascular coupling**			
SPAP, mmHg, median (IQR) (*n* = 47)	30 (29–35)	35 (30–45)	0.027
TAPSE/SPAP ratio, mean ± SD (*n* = 47)	0.7 ± 0.2	0.5 ± 0.2	0.003
TAPSE/SPAP < 0.55, n (%) (*n* = 47)	3 (20)	16 (67)	0.005

Data are presented as mean ± SD, median (IQR), or n (%). LVEF = left ventricular ejection fraction; LV-LWFS = LV longitudinal wall fractional shortening; RV-FAC = right ventricular fractional area change; SPAP = systolic pulmonary artery pressure; TAPSE = tricuspid annular plane systolic excursion; VAC = ventriculo–arterial coupling

Additional associations between CMD and echocardiographic abnormalities among the 49 patients with complete coronary physiological assessment are shown in Table 3. Complete echocardiographic data are presented in Supplementary Table S4. Left ventricular systolic and diastolic function were similar in patients with and without CMD .

In exploratory analyses, elevated IMR was associated with impaired right ventricular systolic function and impaired right ventricular-pulmonary arterial coupling, whereas no consistent associations were observed for reduced MRR (Supplementary Table S5).

No major differences in echocardiographic findings were observed between patients with and without obstructive CAD (Supplementary Table S3).

### Impact of sepsis on coronary microvascular function

**Fig. 4:**
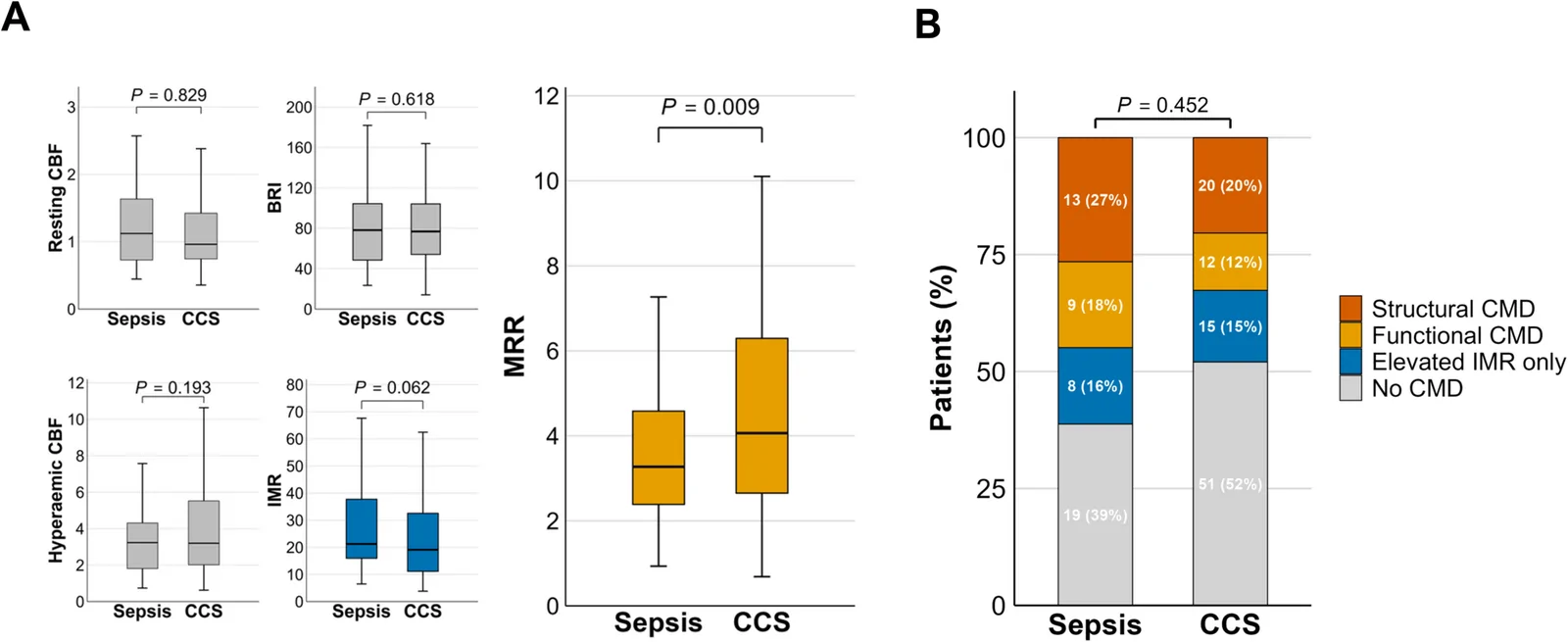
Coronary microvascular function in sepsis and matched chronic coronary syndrome controls. (**A**) Box plots comparing patients with sepsis (*n* = 49) and age-, sex-, and CAD-matched chronic coronary syndrome (CCS) controls (*n* = 98) with respect to resting coronary blood flow (CBF), baseline resistance index (BRI), hyperaemic CBF, index of microcirculatory resistance (IMR), and microvascular resistance reserve (MRR). Boxes represent interquartile ranges with median lines. Brackets indicate pairwise comparisons derived from linear mixed-effects models; corresponding *P* values are shown. (**B**) Distribution of coronary microvascular phenotypes classified as no coronary microvascular dysfunction (CMD), elevated IMR only, functional CMD, or structural CMD. *P* value for the overall comparison of coronary microvascular function phenotypes between the sepsis and CCS groups

Patients with sepsis had lower median MRR values than CCS patients matched for age, sex, and extent of obstructive CAD (Fig. 4A). IMR tended to be higher in patients with sepsis, whereas resting and hyperaemic coronary blood flow and BRI were similar between groups (Fig. 4A). Normal coronary microvascular function was observed less frequently in patients with sepsis than in CCS controls (39% vs. 52%), although this difference did not reach statistical significance (Fig. 4B). Baseline characteristics of the matched cohorts are presented in Supplementary Table S6.

## Discussion

### Principal findings

In this prospective study we performed the first invasive assessment of coronary microvascular function using thermodilution technique in adults with sepsis and myocardial injury. CMD was prevalent and heterogeneous, with phenotypes ranging from isolated increases in hyperaemic microvascular resistance (elevated IMR only) to isolated impairment of vasodilatory capacity (functional CMD) or combined abnormalities (structural CMD). Obstructive CAD was present in approximately one fifth of patients, with all but one case occurring in patients without a history of previous myocardial infarction.

Invasively measured coronary microvascular indices were not associated with either plasma hs-cTnT or NT-proBNP concentrations despite the high prevalence of CMD. hs-cTnT concentrations were associated with left ventricular systolic dysfunction, whereas NT-proBNP was associated with a broader pattern of cardiac and haemodynamic dysfunction. CMD was not associated with conventional measures of left ventricular dysfunction. However, it was associated with ventriculo-arterial uncoupling, impaired right ventricular systolic function, and impaired right ventricular-pulmonary arterial coupling. Patients with sepsis also demonstrated more severely impaired coronary vasodilatory capacity than matched CCS controls, reflected by lower MRR values.

### Coronary microvascular dysfunction is common and heterogeneous in sepsis

The coronary circulation in sepsis has received little direct investigation, probably due to considerable illness severity and technical challenges. The pioneering studies by Cunnion and colleagues and Dhainaut and colleagues in the 1980s used coronary sinus thermodilution techniques to provide the first invasive assessments of coronary physiology in patients with septic shock. These studies used measurements of total coronary venous effluent and demonstrated preserved or increased coronary blood flow together with low calculated vascular resistance, challenging the prevailing concept at the time that myocardial dysfunction in sepsis primarily reflected global coronary hypoperfusion [25, 26]. These techniques provided global assessment of coronary perfusion and could not directly evaluate coronary microvascular function.

More recent studies have suggested that coronary microvascular function may be abnormal during sepsis. In a porcine model of hyperdynamic sepsis, Boudart et al. demonstrated impaired coronary autoregulation, reduced coronary flow reserve (CFR), and increased hyperaemic microvascular resistance despite preserved coronary blood flow, highlighting the importance of directly assessing microvascular physiology rather than relying solely on global measures of coronary perfusion [27]. Human data remain limited. Ikonomidis et al. used transthoracic Doppler echocardiography to demonstrate reduced CFR in patients with sepsis, providing the first evidence of impaired coronary vasodilatory capacity in humans [13].

The present study extends previous observations substantially. Using contemporary pressure- and thermodilution physiology, we performed the first direct characterisation of coronary microvascular function in patients with sepsis and myocardial injury and demonstrated that CMD is common and physiologically heterogeneous. We identified four physiological phenotypes, suggesting that CMD in sepsis is unlikely to reflect a single pathophysiological process.

These phenotypes may provide insight into the mechanisms underlying coronary microvascular dysfunction in sepsis. The functional and structural CMD framework was developed primarily from invasive coronary physiology studies in stable patients with CCS, rather than in acute critical illness populations. In this setting, structural CMD is typically attributed to abnormalities that increase microvascular resistance, such as vascular remodelling, extravascular compression, and luminal obstruction. Functional CMD is typically attributed to impaired vasodilatory function related to endothelial dysfunction, inflammation, oxidative stress, and impaired microvascular autoregulation with elevated resting flow [28, 29].

These classifications may also be relevant in sepsis. However, the underlying pathophysiological mechanisms are likely to differ. Acute inflammation, endothelial injury, tissue oedema with extravascular compression, and microvascular thrombosis may increase microvascular resistance and/or impair vasodilatory capacity. The resulting physiological patterns may therefore resemble those observed in chronic coronary microvascular dysfunction, despite potentially arising through different pathophysiological mechanisms [16].

### Determinants of coronary microvascular dysfunction in sepsis

CMD was associated with higher CRP concentrations, supporting a link between coronary microvascular dysfunction and the systemic inflammatory response central to sepsis. Inflammation in sepsis promotes endothelial activation, glycocalyx degradation, and disturbances in vascular flow regulation, all of which contribute to widespread microvascular injury affecting multiple vascular beds [30]. The association between CRP and CMD therefore provides indirect support for a contribution of these processes to the coronary circulation.

Further support for the concept that CMD in sepsis occurs as part of a broader pattern of cardiovascular and microvascular derangement comes from its association with abnormalities in ventriculo-arterial coupling, right ventricular systolic function, and right ventricular–pulmonary arterial coupling. Coronary microvascular abnormalities would be expected to occur alongside disturbances in systemic vascular function, pulmonary vascular function, and ventricular–vascular interactions in a syndrome characterised by widespread microvascular injury [31].

Patients with CMD also had lower baseline renal function than those without CMD. Chronic kidney disease has previously been associated with impaired coronary microvascular function, even in the absence of obstructive CAD [32]. Lower baseline eGFR may therefore identify patients with pre-existing microvascular vulnerability, in whom CMD in sepsis arises from an acute insult superimposed on chronic microvascular disease.

### Temporal dynamics of coronary microvascular dysfunction in sepsis

Coronary angiography and invasive physiological assessment were performed following clinical stabilisation, a mean of 4 days after sepsis onset. Consequently, our findings should be interpreted as reflecting coronary microvascular function during the subacute phase of sepsis rather than during the initial haemodynamic insult. It is plausible that both the prevalence and severity of CMD are greater during the acute phase of sepsis than observed in our cohort.

Whether the abnormalities identified represent persistent injury, recovery from an earlier and more severe insult, or pre-existing microvascular disease remains uncertain. Experimental studies suggest that CMD may persist beyond resolution of the acute inflammatory response, while observations from other vascular beds indicate that microvascular abnormalities evolve dynamically during sepsis and recovery [25, 27, 33]. The CMD phenotypes observed in our cohort may therefore represent both distinct physiological entities and different stages of a dynamic process.

The reversibility of CMD following sepsis remains uncertain. Acute endothelial and microvascular injury during sepsis may initiate more persistent structural alterations of the coronary microcirculation, potentially contributing to long-term microvascular dysfunction even after resolution of the acute illness [34–36]. Equally, some abnormalities may be transient and reversible [37]. In a previously reported case from this cohort, repeat physiological assessment 30 days after sepsis onset demonstrated normalisation of marked CMD observed during sub-acute illness [38]. This finding is hypothesis-generating but suggests that some abnormalities are reversible and highlights the need for serial physiological studies during recovery.

### Coronary microvascular dysfunction was not associated with myocardial injury

CMD may impair myocardial oxygen delivery and contribute to supply-demand mismatch. We therefore hypothesised that it would be an important determinant of troponin release in sepsis [15]. However, hs-cTnT concentrations were not associated with either IMR or MRR, and restricted cubic spline analyses demonstrated no evidence of linear or non-linear relationships.

CMD and troponin elevation were both common in our cohort. However, hs-cTnT concentrations were unrelated to the presence or severity of measurable microvascular impairment. These findings suggest that CMD and cardiomyocyte injury represent parallel manifestations of cardiac involvement in sepsis rather than a direct cause-and-effect relationship. Both may arise from common upstream processes. Myocardial injury in sepsis likely reflects a complex interplay between inflammatory injury, metabolic and oxidative stress, abnormalities in ventricular loading conditions, and, in selected patients, CAD and/or CMD. These findings are consistent with previous studies linking troponin elevation to cardiac dysfunction in sepsis and support the concept that troponin release reflects cardiomyocyte injury occurring within the broader syndrome of sepsis-induced myocardial dysfunction [39, 40]. The multifactorial nature of myocardial injury in sepsis may therefore help explain the absence of a direct association between coronary microvascular indices, troponin concentrations, and measures of cardiac dysfunction in the present study. Furthermore, myocardial injury and cardiac dysfunction may arise through mechanisms other than impaired coronary perfusion, including abnormalities in oxygen utilisation and cellular metabolism [41, 42]. This may explain why neither troponin concentrations nor cardiac dysfunction were associated with coronary physiological indices in the present study.

### Obstructive CAD in sepsis with myocardial injury

Obstructive CAD was identified in 22% of patients, despite only one patient having a documented history of previous myocardial infarction. This suggests that clinically relevant coronary disease may frequently remain unrecognised in patients presenting with sepsis-associated myocardial injury. At present, there are no guidelines regarding the investigation of underlying CAD in patients with sepsis-related troponin elevation, and decisions regarding further cardiovascular evaluation are therefore largely left to clinician judgement. Consistent with this, patients with sepsis and suspected myocardial infarction appear less likely to undergo coronary angiography and revascularisation than patients presenting with myocardial infarction in the absence of sepsis [43].

Most patients had no obstructive CAD and exhibited either normal coronary arteries or only mild non-obstructive atherosclerosis. These findings suggest that although obstructive CAD represents an important and potentially treatable comorbidity in some patients with sepsis and myocardial injury, it is unlikely to be the predominant cause of troponin elevation in this setting. No patients in our cohort had suspected type 1 myocardial infarction; nevertheless, underlying obstructive CAD may have contributed to myocardial injury in selected patients by increasing their vulnerability to myocardial oxygen supply-demand mismatch during acute illness. Because previous coronary imaging studies in sepsis have largely been restricted to highly selected patients with suspected acute coronary syndromes, the prevalence and contribution of obstructive CAD to myocardial injury in broader sepsis populations remain poorly defined [6–8].

Our findings do not support routine coronary angiography in patients with sepsis and myocardial injury. However, patients with obstructive CAD exhibited a more traditional cardiovascular risk profile, suggesting that established clinical markers of coronary artery disease remain informative even in the setting of sepsis-associated myocardial injury. Obstructive CAD was unlikely to represent the predominant mechanism of myocardial injury. Nevertheless, its identification often resulted in clinically actionable management, including revascularisation and/or optimisation of preventive cardiovascular therapy. These findings suggest that selective evaluation may identify previously unrecognised and potentially treatable coronary artery disease.

### Strengths and limitations

A major strength of this study is its prospective design with consecutive patient enrolment and protocolised data collection. The use of invasive coronary angiography combined with detailed thermodilution-derived assessment of coronary microvascular function provides physiological insights not previously available in this population.

Some limitations should be acknowledged. Firstly, the requirement for informed consent and sufficient clinical stability to undergo coronary angiography excluded the most severely ill patients, and the findings may therefore not be generalisable to all patients with sepsis.

Second, coronary physiological assessment was performed approximately four days after sepsis onset and plausibly after peak hs-cTnT concentrations had occurred. As coronary microvascular function is dynamic, partial recovery may have attenuated associations between biomarkers and physiological indices. Nevertheless, assessment following clinical stabilisation reduced potential confounding from acute haemodynamic instability and vasoactive therapy.

Third, pre-existing CMD could not be determined, limiting the ability to distinguish sepsis-induced abnormalities from antecedent microvascular disease. Fourth, the prevalence of diabetes mellitus was higher in the sepsis cohort than in the matched CCS cohort and residual confounding cannot be excluded. Finally, adenosine-induced hyperaemia primarily assesses non-endothelium-dependent coronary microvascular function and does not capture endothelial-dependent abnormalities.

## Conclusions

CMD was common and physiologically heterogeneous in patients with sepsis and myocardial injury. Invasive measures of coronary microvascular function were not associated with troponin concentrations, suggesting that CMD and myocardial injury may represent parallel but not directly coupled manifestations of cardiac involvement in sepsis. Previously unrecognised obstructive CAD was identified in approximately one fifth of patients, supporting consideration of underlying obstructive CAD in patients with sepsis and troponin elevation.

## Supplementary Information

Below is the link to the electronic supplementary material.

Supplementary Material 1.

## Data Availability

The datasets used and/or analysed during the current study are available from the corresponding author on reasonable request.

## Abbreviations

BRI: Baseline Resistance Index
CAD: Coronary artery disease
CBF: Coronary blood flow
CCS: Chronic coronary syndrome
CMD: Coronary microvascular dysfunction
CRP: C-reactive protein
FFR: Fractional flow reserve
hs-cTnT: High-sensitivity cardiac troponin T
IMR: Index of microcirculatory resistance
LAD: Left anterior descending coronary artery
ln: Natural logarithm
LVEF: Left ventricular ejection fraction
MRR: Microvascular resistance reserve
NT-ProBNP: N-terminal pro–B-type natriuretic peptide
PCI: Percutaneous coronary intervention
RV-FAC: Right ventricular fractional area change
SPAP: Systolic pulmonary artery pressure
TAPSE: Tricuspid annular plane systolic excursion
TTE: Transthoracic echocardiography
VAC: Ventriculo-arterial coupling
