# Correction to: Coronary microvascular function in patients with sepsis and myocardial injury: an invasive coronary physiology study

**DOI:** 10.1186/s13054-026-06298-x

**Published:** 2026-09-18

**Authors:** Samantha Lörstad, Per Åstrand, Patrik Gille-Johnson, Yunzhang Wang, Christina Ekenbäck, Fadi Jokhaji, Felix Böhm, Patrik Hjalmarsson, Shajan Shekarestan, Tomas Jernberg, Sara Tehrani, Kambiz Shahgaldi, Jonas Persson

**Affiliations:** 1 Department of Clinical Sciences, Division of Internal Medicine, Karolinska Institutet, Danderyd University Hospital, Stockholm, Sweden; 2 Internal Medicine Clinic, Danderyd University Hospital, Stockholm, Sweden; 3 Infectious Diseases Clinic, Danderyd University Hospital, Stockholm, Sweden; 4 Department of Clinical Sciences, Karolinska Institutet, Danderyd University Hospital, Stockholm, Sweden; 5 Department of Clinical Sciences, Division of Cardiovascular Medicine, Karolinska Institutet, Danderyd University Hospital, Stockholm, Sweden

**Correction to: Crit Care 30, 351 (2026).**

**https://doi.org/10.1186/s13054-026-06179-3**.

Following publication of the original article [1], the authors identified errors in the Data availability section and in Table 3. The data availability section at the end of the article was incorrect. In Table 3 the values within the variable Left ventricular diastolic function weren’t correctly aligned. Both the incorrect and correct information is given hereafter.

The incorrect Data availability section:

**Data availability**

No datasets were generated or analysed during the current study.

The correct Data availability section:

**Data availability**

The datasets used and/or analysed during the current study are available from the corresponding author on reasonable request.

The incorrect Table 3:

**Table 3:** Echocardiographic characteristics according to the presence of coronary microvascular dysfunction (*n* = 49)

Variable	No CMD (*n* = 19)	CMD (*n* = 30)	*P*
**Left ventricular systolic function**			
Cardiac output, L/min, mean ± SD	5.8 ± 1.3	5.6 ± 1.5	0.46
LVEF, %, median (IQR)	57 (45–62)	51 (45–59)	0.417
Regional wall motion abnormality, n (%)	7 (37)	7 (24)	0.344
LV-LWFS, %, median (IQR)	12 (10–14)	12 (10–14)	0.591
**Ventriculo-arterial coupling**			
VAC > 1.0, n (%)	9 (47)	23 (78)	0.036
**Left ventricular diastolic function**			
E/e´ ratio, median (IQR) (*n* = 47)	10 (8–12)	10 (8–12)	0.86
Diastolic function classification, n (%) (*n* = 48) - Normal diastolic function - Impaired relaxation with normal filling pressures - Impaired relaxation with elevated filling pressures	10 (53) 4 (21) 5 (26)	8 (28) 6 (21) 15 (52)	0.157
**Right ventricular systolic function**			
TAPSE, mm, mean ± SD	21 ± 5	18 ± 4	0.008
RV-FAC, %, mean ± SD	38 ± 8	32 ± 7	0.012
RV-FAC < 35%, n (%)	5 (29)	18 (69)	0.01
**Right ventricular-pulmonary vascular coupling**			
SPAP, mmHg, median (IQR) (*n* = 47)	30 (29–35)	35 (30–45)	0.027
TAPSE/SPAP ratio, mean ± SD (*n* = 47)	0.7 ± 0.2	0.5 ± 0.2	0.003
TAPSE/SPAP < 0.55, n (%) (*n* = 47)	3 (20)	16 (67)	0.005

Data are presented as mean ± SD, median (IQR), or n (%). LVEF = left ventricular ejection fraction; LV-LWFS = LV longitudinal wall fractional shortening; RV-FAC = right ventricular fractional area change; SPAP = systolic pulmonary artery pressure; TAPSE = tricuspid annular plane systolic excursion; VAC = ventriculo–arterial coupling

The correct Table 3:

**Table 3:** Echocardiographic characteristics according to the presence of coronary microvascular dysfunction (*n* = 49)

Variable	No CMD (*n* = 19)	CMD (*n* = 30)	*P*
**Left ventricular systolic function**			
Cardiac output, L/min, mean ± SD	5.8 ± 1.3	5.6 ± 1.5	0.46
LVEF, %, median (IQR)	57 (45–62)	51 (45–59)	0.417
Regional wall motion abnormality, n (%)	7 (37)	7 (24)	0.344
LV-LWFS, %, median (IQR)	12 (10–14)	12 (10–14)	0.591
**Ventriculo-arterial coupling**			
VAC > 1.0, n (%)	9 (47)	23 (78)	0.036
**Left ventricular diastolic function**			
E/e´ ratio, median (IQR) (*n* = 47)	10 (8–12)	10 (8–12)	0.86
Diastolic function classification, n (%) (*n* = 48)			
- Normal diastolic function	10 (53)	8 (28)	0.157
- Impaired relaxation with normal filling pressures	4 (21)	6 (21)	
- Impaired relaxation with elevated filling pressures	5 (26)	15 (52)	
**Right ventricular systolic function**			
TAPSE, mm, mean ± SD	21 ± 5	18 ± 4	0.008
RV-FAC, %, mean ± SD	38 ± 8	32 ± 7	0.012
RV-FAC < 35%, n (%)	5 (29)	18 (69)	0.01
**Right ventricular-pulmonary vascular coupling**			
SPAP, mmHg, median (IQR) (*n* = 47)	30 (29–35)	35 (30–45)	0.027
TAPSE/SPAP ratio, mean ± SD (*n* = 47)	0.7 ± 0.2	0.5 ± 0.2	0.003
TAPSE/SPAP < 0.55, n (%) (*n* = 47)	3 (20)	16 (67)	0.005

Data are presented as mean ± SD, median (IQR), or n (%). LVEF = left ventricular ejection fraction; LV-LWFS = LV longitudinal wall fractional shortening; RV-FAC = right ventricular fractional area change; SPAP = systolic pulmonary artery pressure; TAPSE = tricuspid annular plane systolic excursion; VAC = ventriculo–arterial coupling

The data availability section and Table 3 have been updated in this correction article and the original article [1] has been corrected.
