# Supplementary material for: Coronary microvascular function in patients with sepsis and myocardial injury: an invasive coronary physiology study
Source: Crit Care. 2026 Jul 2;30:351. doi: 10.1186/s13054-026-06179-3 (PMC13330245; doi:10.1186/s13054-026-06179-3)
Supplement: Supplementary file 1 — Supplementary Material 1. [file 13054_2026_6179_MOESM1_ESM.docx]

**Supplementary Material**

**Contents**

**Supplementary Methods (1)** Patient Eligibility **............................................................... 2**

**Supplementary Methods (2)** Echocardiographic Assessment **.......................................... 4**

**Supplementary Table S1.** Coronary Flow and Microcirculatory Resistance Indices: Definitions and Formulas **.................................................................................................... 7**

**Supplementary Table S2.** Coronary microvascular function according to the presence of obstructive coronary artery disease **..................................................................................... 8**

**Supplementary Table S3.** Echocardiographic characteristics in patients undergoing coronary angiography according to the presence of obstructive coronary artery disease **.................. 9**

**Supplementary Table S4.** Echocardiographic characteristics according to coronary microvascular dysfunction status (n = 49) **........................................................................... 12**

**Supplementary Table S5.** Associations of echocardiographic abnormalities with IMR and MRR in patients undergoing coronary microvascular assessment (n=49) **........................... 14**

**Supplementary Table S6.** Baseline characteristics of the sepsis and CCS cohorts **........... 15**

**Supplementary Figure S1.** Extended patient flow chart **................................................... 16**

**Supplementary Figure S2.** Distribution of cardiac biomarkers **........................................ 18**

**Supplementary Figure S3.** Associations between cardiac biomarkers and echocardiographic indices in sepsis **................................................................................................................... 19**

**Supplementary References ............................................................................................... 20**

**Supplementary Methods**

**(1) Patient Eligibility**

Eligible patients were:

- treated for sepsis or septic shock according to the Sepsis-3 criteria
- aged 40–85 years
- had an expected survival >1 year
- demonstrated myocardial injury, defined as a plasma hs-cTnT concentration ≥15 ng/L within 48 hours of sepsis onset

All participants provided written informed consent

Patients were screened on the basis of sepsis-associated myocardial injury, irrespective of symptoms suggestive of acute coronary syndrome or prior suspicion of coronary artery disease.

Pre-specified exclusion criteria were:

- pregnancy
- previous coronary artery bypass grafting
- prior heart transplantation
- pre-admission left ventricular (LV) ejection fraction ≤39%
- hypertrophic cardiomyopathy (septal thickness >15 mm)
- severe aortic stenosis
- cardiac amyloidosis or sarcoidosis with myocardial involvement
- chronic kidney disease with a pre-admission estimated glomerular filtration rate (eGFR) <30 mL/min/1.73 m²
- asthma
- infective endocarditis
- major abdominal, thoracic, or orthopaedic surgery within three months before admission

In addition, patients were not eligible if they:

- had persistent clinical instability beyond day 8 (ongoing circulatory shock requiring vasopressors or respiratory failure requiring invasive or non-invasive ventilation)
- had a mechanical aortic valve or prior transcatheter aortic valve implantation
- had severe mitral valve disease
- unresolved acute kidney failure by day 10 (eGFR <45 mL/min/1.73 m²)
- were transferred to another hospital before inclusion
- had contraindications to potential downstream coronary revascularisation or guideline-directed medical therapy should clinically significant obstructive CAD be identified on angiography (e.g., inability to receive antiplatelet or anticoagulant therapy).

These criteria were applied pragmatically for safety, ethical, and feasibility reasons.

**(2) Echocardiographic Assessment**

**Equipment and image acquisition**

Transthoracic echocardiography was performed using Vivid E9 or Vivid E95 ultrasound systems (GE Healthcare, Horten, Norway) equipped with an M5Sc transducer. Standard parasternal and apical views were acquired with patients in the supine or left lateral decubitus position, as clinically feasible.

All examinations were performed by certified and experienced sonographers or clinical physiologists. Offline analysis was conducted by a single certified assessor who was blinded to clinical characteristics and invasive coronary physiology data to ensure quality and consistency.

**Left ventricular size and systolic function**

Left ventricular (LV) dimensions and volumes were measured in accordance with current American Society of Echocardiography and European Association of Cardiovascular Imaging recommendations.(1) LV ejection fraction was calculated using the biplane Simpson method.(1)

LV longitudinal systolic function was assessed using longitudinal wall fractional shortening (LV-LWFS) from the apical four-chamber (A4C) view.(2) Mitral annular plane systolic excursion (MAPSE) was measured as the longitudinal displacement of the mitral annulus from end-diastole to end-systole using M-mode or two-dimensional imaging. In the A4C view, MAPSE was obtained at the septal and lateral mitral annular insertion points, and the mean of these measurements was used for analysis. LV end-diastolic length was measured in the same A4C view as the distance from the midpoint of the mitral annular plane to the apical endocardium at end-diastole. LV-LWFS was calculated as MAPSE divided by LV end-diastolic length and expressed as a percentage.(2)

Speckle-tracking–derived global longitudinal strain (GLS) could not be obtained in the majority of patients owing to inadequate image quality for strain analysis in the critical care setting.(3) LV-LWFS was therefore selected as a pragmatic and robust surrogate measure of LV longitudinal systolic function and has been applied in previous studies of critically ill patients.(3-5) Values >12% were considered normal.(4, 5) Regional wall motion abnormalities were assessed visually using the standard 17-segment model.(1)

**LV contractility and left ventricular–arterial coupling**

LV contractility was quantified as end-systolic elastance (Ees), estimated non-invasively using a validated single-beat method as described by Chen *et al.*, which incorporates echocardiographically derived left ventricular end-systolic and end-diastolic volumes together with contemporaneous systolic and diastolic blood pressure measurements.(6)

Global cardiovascular performance, reflecting the interaction between LV systolic function and net arterial load, was assessed using ventriculo–arterial coupling (VAC). Effective arterial elastance (Ea) was calculated as the ratio of end-systolic pressure to stroke volume; end-systolic pressure was estimated as 0.9 × brachial systolic blood pressure measured contemporaneously.(7) Stroke volume was derived from echocardiographic measurements. VAC was defined as the ratio of Ea to Ees.(7)

**Left ventricular diastolic function**
LV diastolic function was assessed and categorised in accordance with British Society of Echocardiography guidelines.(8) Assessment integrated transmitral inflow velocities (E and A waves), tissue Doppler imaging of the mitral annulus (septal and lateral e′ velocities), left atrial volume index (LAVi), and tricuspid regurgitation peak velocity when available. Diastolic function was classified as normal, abnormal with normal left atrial pressure, or abnormal with elevated left atrial pressure, using an integrative approach incorporating multiple parameters rather than any single measurement.(8)

**Right ventricular function and right ventricular–pulmonary artery coupling**

Right ventricular (RV) size and systolic function were evaluated in accordance with American Society of Echocardiography recommendations for right heart assessment.(9) RV systolic function was assessed using M-mode–derived tricuspid annular plane systolic excursion (TAPSE) and right ventricular fractional area change (RV-FAC). RV afterload was estimated by systolic pulmonary artery pressure (SPAP), calculated from the peak tricuspid regurgitation velocity and right atrial pressure derived from inferior vena cava diameter and collapsibility.

RV–pulmonary artery coupling was assessed using the TAPSE/SPAP ratio, which was used as an index of the relationship between RV contractile function and pulmonary arterial load.(10)

| **Table S1. Coronary Flow and Microcirculatory Resistance Indices: Definitions and Formulas** | | |
| --- | --- | --- |
| **Flow Index** | **Formula** | **Explanation** |
| Resting coronary blood flow (CBF) ^(11)^ | 1 / Tmn_rest | Estimated resting flow velocity. |
| Baseline resistance index (BRI)(12) | Pd_rest × Tmn_rest | Resting microvascular resistance. |
| Hyperaemic coronary blood flow (CBF) | 1 / Tmn_hyp | Estimated hyperaemic flow velocity |
| Corrected IMR(13) | Pa_hyp × Tmn_hyp × (1.35 ×  Pd_hyp / Pa_hyp – 0.32) | Hyperaemic microvascular resistance corrected for collateral flow. |
| Fractional flow reserve (FFR)(14) | Pd_hyp / Pa_hyp | Distal to aortic pressure ratio during hyperaemia |
| Coronary flow reserve (CFR)(12) | Tmn_rest / Tmn_hyp | Vasodilatory capacity of the coronary circulation |
| Microvascular resistance reserve (MRR)(15) | (CFR / FFR) × (Pa_rest / Pa_hyp) | Vasodilatory capacity corrected for epicardial stenosis |
| **Abbreviations:** Pa_hyp = aortic pressure during hyperaemia; Pa_rest = aortic pressure during rest; Pd_hyp = distal coronary pressure during hyperaemia; Pd_rest = distal coronary pressure during rest; Tmn_hyp = mean transit time of a saline bolus during hyperaemia; Tmn_rest = mean transit time of a saline bolus during rest. | | |

| **Table S2. Coronary microvascular function according to the presence of obstructive coronary artery disease** | | | |
| --- | --- | --- | --- |
| **Coronary microvascular indices** | **Total**  **(n = 49)** | **No CAD**  **(n = 40)** | **CAD***  **(n = 8)** |
| Resting CBF, median (IQR) | 1.1 (0.7–1.6) | 1.0 (0.7–1.5) | 1.5 (0.8–2.4) |
| BRI, median (IQR) | 78 (48–105) | 82 (52–108) | 44 (31–83) |
| Hyperaemic CBF, median (IQR) | 3.2 (1.8–4.3) | 2.9 (1.8–4.2) | 3.4 (2.1–5.2) |
| IMR, median (IQR) | 21 (15–38) | 24 (17–40) | 15 (13–32) |
| IMR > 25, n (%) | 22 (45) | 20 (50) | 2 (25) |
| MRR, median (IQR) | 3.3 (2.2–4.8) | 3.4 (2.0–4.9) | 3.0 (2.2–5.0) |
| MRR ≤ 3.0, n (%) | 22 (45) | 17 (43) | 4 (50) |
| CMD, n (%)† | 30 (61) | 24 (60) | 5 (62) |
| * Coronary angiography was performed in 55 patients, and coronary microvascular indices were measured in 49/55 patients. One patient with obstructive CAD did not undergo coronary microvascular assessment and was therefore excluded from CAD subgroup analyses; consequently, subgroup denominators do not sum to the denominator in the overall column.  † CMD was defined as MRR ≤3 and/or IMR >25.  **Abbreviations.** BRI = baseline resistance index; CBF = coronary blood flow; CMD = coronary microvascular dysfunction; FFR = fractional flow reserve; IMR = index of microcirculatory resistance; LVEDP = left ventricular end-diastolic pressure; MRR = microvascular resistance reserve. | | | |

| **Table S3. Echocardiographic characteristics in patients undergoing coronary angiography according to the presence of obstructive coronary artery disease** | | | |
| --- | --- | --- | --- |
| **Variable** | **Total**  **(n = 55)** | **No obstructive CAD**  **(n = 43)** | **CAD**  **(n = 12)** |
| **Left ventricular systolic function** |  |  |  |
| Cardiac output, L/min, mean ± SD | 5.6 ± 1.4 | 5.7 ± 1.4 | 5.5 ± 1.0 |
| LVESV, mL, median (IQR)  Men  Women (n = 1) | 60 (40–76)  46 (34–67) | 59 (41–74)  46 (33–65) | 65 (40–104)  - |
| LVEDV, mL, median (IQR)  Men  Women (n = 1) | 125 (107–165)  107 (83–117) | 126 (114–162)  121 (103–173) | 107 (82–115)  - |
| LVEF, %, median (IQR)  Men  Women (n = 1) | 55 (47–60)  56 (42–59) | 56 (48–60)  47 (41–61) | 57 (44–59)  - |
| Regional wall motion abnormality, n (%) | 14 (26) | 8 (20) | 5 (42) |
| LV-LWFS, %, median (IQR) | 12 (10–14) | 12 (11–14) | 11 (9–14) |
| MAPSE, mm, mean ± SD | 10.1 ± 2.3 | 10.4 ± 2.2 | 9.1 ± 2.7 |
| LV elastance, mmHg/mL, median (IQR) | 1.3 (1.2–1.7) | 1.3 (1.2–1.7) | 1.5 (1.1–1.9) |
| VAC, median (IQR) | 1.1 (1.0–1.4) | 1.1 (1.0–1.4) | 1.2 (1.0–1.3) |
| VAC >1.0, n (%) | 36 (66) | 26 (62) | 9 (75) |
|  |  |  |  |
| **Left ventricular diastolic function** | |  |  |
| E-wave velocity, m/s, mean ± SD (*n*=53) | 0.7 ± 0.2 | 0.8 ± 0.2 | 0.9 ± 0.2 |
| E/A ratio, median (IQR) (*n*=53) | 1.0 (0.7–1.2) | 1.0 (0.7–1.2) | 1.0 (0.7–1.4) |
| Septal e´, cm/s, median (IQR) (*n*=53) | 7 (5–9) | 7 (5–9) | 6 (4–9) |
| Lateral e´, cm/s, mean ± SD (*n*=53) | 9.3 ± 2.8 | 9.6 ± 2.8 | 8.1 ± 2.5 |
| E/e´ ratio, median (IQR) (*n*=53) | 10 (8–12) | 9 (7–12) | 12 (8–15) |
| LAVi, mL/m^2^, mean ± SD (*n*=47) | 37 ± 9.9 | 36 ± 9.6 | 39 ± 10.1 |
| SPAP, mmHg, median (IQR) (*n*=47) | 35 (30–40) | 35 (30–40) | 35 (30–41) |
| Diastolic function classification, n (%) (n=54)  - Normal diastolic function  - Impaired relaxation with normal filling pressures  - Impaired relaxation with elevated filling pressures | 22 (40)  10 (18)  22 (40) | (n = 41)  19 (46)  7 (17)  15 (37) | 3 (25)  2 (17)  7 (58) |
|  |  |  |  |
| **Right ventricular dimensions** |  |  |  |
| RVDd, mm, mean ± SD | 40 ± 5.7 | 40 ±5.9 | 38 ± 4.9 |
| RVDd >42 mm, n (%) | 19 (35) | 15 (36) | 3 (25) |
|  |  |  |  |
| **Right ventricular systolic function** | |  |  |
| TAPSE, mm, mean ± SD | 19 ± 5 | 19 ± 5 | 20 ± 4 |
| TAPSE/SPAP ratio, mean ± SD (*n*=47) | 0.6 ± 0.2 | 0.6 ± 0.2 | 0.6 ± 0.2 |
| TAPSE/SPAP < 0.55, n (%) (*n*=47) | 22 (40) | 18 (51) | 4 (44) |
| TAPSE/SPAP < 0.33, n (%) (*n*=47) | 5 (9) | 5 (14) | 0 (0) |
| RV-FAC, %, mean ± SD | 35 ± 8 | 36 ± 8 | 31 ± 7 |
| RV-FAC <35%, n (%) | 26 (47) | 19 (50) | 7 (70) |
| **Footnotes (normal reference values and definitions).** Cardiac output: 4–6 L/min; E-wave velocity: 41–65 years >0.5 m/s, >65 years >0.4 m/s; E/A ratio: 41–65 years >0.7, >65 years >0.5; E/e′ ratio: ≤14; Lateral e′: 41–65 years >6.0 cm/s, >65 years >5.0 cm/s; Septal e′: 41–65 years >5.0 cm/s, >65 years >4.0 cm/s; LAVi: <35 mL/m²; LVEDV: men 62-150 mL, women 46–106 mL; LVESV: men 21–61 mL, women 14–42 mL; LVEF: men 52–72%, women 54–74%), mildly abnormal (men 41–51%, women 41–53%), moderately abnormal (30–40% for both sexes), and severely abnormal (<30% for both sexes); LV elastance (Ees): <1.0 mmHg/mL; LV-LWFS: >12%; MAPSE: ≥12 mm; RVDd: ≤42 mm; RV-FAC: ≥35%; SPAP: ≤30 mmHg; TAPSE: ≥17 mm; TAPSE/SPAP: 0.8–1.8; VAC: 0.5–1.0.  **Abbreviations.** E/A ratio = early (E) to late (A) diastolic transmitral flow velocity ratio; IMR = index of microcirculatory resistance; LAVi = left atrial volume index; LVEDP = left ventricular end-diastolic pressure; LVEDV = left ventricular end-diastolic volume; LVEF = left ventricular ejection fraction; LVESV = left ventricular end-systolic volume; LV-LWFS = LV longitudinal wall fractional shortening; MAPSE = mitral annular plane systolic excursion; RVDd = right ventricular diastolic diameter; RV-FAC = right ventricular fractional area change; SPAP = systolic pulmonary artery pressure; TAPSE = tricuspid annular plane systolic excursion; VAC = ventriculo–arterial coupling. | | | |

| **Table S4. Echocardiographic characteristics according to coronary microvascular dysfunction status (n = 49)** | | | | |
| --- | --- | --- | --- | --- |
| **Variable** | | | **No CMD**  **(n = 19)** | **CMD**  **(n = 30)** |
| **Left ventricular systolic function** | | |  |  |
| Cardiac output, L/min, mean ± SD | | | 5.8 ± 1.3 | 5.6 ± 1.5 |
| LVESV, mL, median (IQR)  Men  Women | | | 58 (37–73)  59 (45–72) | 62 (48–79)  45 (35–69) |
| LVEDV, mL, median (IQR)  Men  Women | | | 121 (95–158)  95 (82–108) | 126 (109–162)  108 (88–117) |
| LVEF, %, median (IQR)  Men  Women | | | 58 (46–62)  39 (33–45) | 50 (47–60)  58 (41–59) |
| Regional wall motion abnormality, n (%) | | | 7 (37) | 7 (24) |
| LV-LWFS, %, median (IQR) | | | 12 (10–14) | 12 (10–14) |
| MAPSE, mm, mean ± SD | | | 10.5 ± 2.8 | 9.7 ± 2.0 |
| LV elastance, mmHg/mL, median (IQR) | | | 1.3 (1.2–1.9) | 1.4 (1.1–1.7) |
| VAC, median (IQR) | | | 1.0 (0.8–1.3) | 1.0 (0.9–1.3) |
| VAC >1.0, n (%) | | | 9 (47) | 23 (78) |
|  | | |  |  |
|  |  |  |  |  |
| E-wave velocity, m/s, mean ± SD (n = 43) | | | 0.8 ± 0.1 | 0.8 ± 0.2 |
| E/A ratio, median (IQR) (n=38) | | | 0.9 (0.7–1.3) | 1.0 (0.7–1.2) |
| Septal e´, cm/s, median (IQR) (n=48) | | | 6 (5–8) | 7 (5–10) |
| Lateral e´, cm/s, mean ± SD (n=48) | | | 9.1 ± 2.9 | 9.2 ± 2.9 |
| E/e´ ratio, median (IQR) (n=47) | | | 10 (8–12) | 10 (8–12) |
| LAVi, mL/m^2^, mean ± SD (n=48) | | | 36 ± 8.8 | 37 ± 10.5 |
| SPAP, mmHg, median (IQR) (n=42) | | | 30 (29–35) | 35 (30–45) |
| Diastolic function classification, n (%) (n=48)  - Normal diastolic function  - Impaired relaxation with normal filling pressures  - Impaired relaxation with elevated filling pressures | | | 10 (53)  4 (21)  5 (26) | 8 (28)  6 (21)  15 (52) |
|  | | |  |  |
| **Right ventricular dimensions** | | |  |  |
| RVDd, mm, mean ± SD | | | 38 ± 5.5 | 41 ± 5.5 |
| RVDd >42 mm, n (%) | | | 5 (26) | 11 (37) |
|  | | |  |  |
|  |  |  |  |  |
| TAPSE, mm, mean ± SD | | | 21 ± 5 | 18 ± 4 |
| TAPSE/SPAP ratio, mean ± SD (*n*=47) | | | 0.7 ± 0.2 | 0.5 ± 0.2 |
| TAPSE/SPAP < 0.55, n (%) (*n*=47) | | | 3 (20) | 16 (67) |
| TAPSE/SPAP < 0.33, n (%) (*n*=47) | | | 0 (0) | 5 (21) |
| RV-FAC, %, mean ± SD | | | 38 ± 8 | 32 ± 7 |
| RV-FAC <35%, n (%) | | | 5 (29) | 18 (69) |
| **Footnotes (normal reference values and definitions).** Cardiac output: 4–6 L/min; E-wave velocity: 41–65 years >0.5 m/s, >65 years >0.4 m/s; E/A ratio: 41–65 years >0.7, >65 years >0.5; E/e′ ratio: ≤14; Lateral e′: 41–65 years >6.0 cm/s, >65 years >5.0 cm/s; Septal e′: 41–65 years >5.0 cm/s, >65 years >4.0 cm/s; LAVi: <35 mL/m²; LVEDV: men 62-150 mL, women 46–106 mL; LVESV: men 21–61 mL, women 14–42 mL; LVEF: men 52–72%, women 54–74%), mildly abnormal (men 41–51%, women 41–53%), moderately abnormal (30–40% for both sexes), and severely abnormal (<30% for both sexes); LV elastance (Ees): <1.0 mmHg/mL; LV-LWFS: >12%; MAPSE: ≥12 mm; RVDd: ≤42 mm; RV-FAC: ≥35%; SPAP: ≤30 mmHg; TAPSE: ≥17 mm; TAPSE/SPAP: 0.8–1.8; VAC: 0.5–1.0.  **Abbreviations:** E/A ratio = early (E) to late (A) diastolic transmitral flow velocity ratio; IMR = index of microcirculatory resistance; LAVi = left atrial volume index; LVEDP = left ventricular end-diastolic pressure; LVEDV = left ventricular end-diastolic volume; LVEF = left ventricular ejection fraction; LVESV = left ventricular end-systolic volume; LV-LWFS = LV longitudinal wall fractional shortening; MAPSE = mitral annular plane systolic excursion; RVDd = right ventricular diastolic diameter; RV-FAC = right ventricular fractional area change; SPAP = systolic pulmonary artery pressure; TAPSE = tricuspid annular plane systolic excursion; VAC = ventriculo–arterial coupling. | | | | |

| **Table S5. Associations of echocardiographic abnormalities with IMR and MRR in patients undergoing coronary microvascular assessment (n = 49)** | | |
| --- | --- | --- |
| **Variable** | **IMR > 25 *P*** | **MRR ≤ 3 *P*** |
| Cardiac output | 0.473 | 0.717 |
| LVEF | 0.840 | 0.526 |
| Regional wall motion abnormality | 0.591 | 0.030 |
| LV-LWFS | 0.892 | 0.126 |
| VAC > 1.0 | 0.166 | 0.519 |
| E/e´ratio | 0.821 | 0.561 |
| Diastolic dysfunction | 0.761 | 0.214 |
| TAPSE | 0.022 | 0.167 |
| RV-FAC | 0.002 | 0.104 |
| RV-FAC <35% | 0.003 | 0.107 |
| SPAP | 0.388 | 0.159 |
| TAPSE/SPAP | 0.017 | 0.296 |
| TAPSE/SPAP < 0.55 | 0.038 | 0.265 |
| P-values derived from univariable comparisons between patients with normal and abnormal MRR (≤3) or IMR (>25).  **Abbreviations:**  IMR = index of microcirculatory resistance; LVEF = left ventricular ejection fraction; LV-LWFS = LV longitudinal wall fractional shortening; RV-FAC = right ventricular fractional area change; SPAP = systolic pulmonary artery pressure; TAPSE = tricuspid annular plane systolic excursion; VAC = ventriculo–arterial coupling. | | |

| **Table S6. Baseline characteristics of the sepsis and CCS cohorts** | | |
| --- | --- | --- |
|  | **Sepsis cohort**  **(*n*=49)** | **CCS cohort**  **(*n*=98)** |
| **Demographics** |  |  |
| Age, years, median (IQR) | 74 (69–79) | 75 (70–79) |
| Female sex | 15 (31) | 30 (31) |
| Current or former smoker | 29 (59) | 63 (64) |
| BMI, kg/m^2^, median (IQR) | 25 (24–29) | 26 (24–29) |
|  |  |  |
| **Coronary vessels with obstructive CAD** |  |  |
| Single-vessel disease  Two-vessel disease  Main stem only  Main stem + two vessels | 5 (10)  5 (10)  1 (2)  1 (2) | 10 (10)  10 (10)  2 (2)  2 (2) |
|  |  |  |
| **Comorbid history** |  |  |
| Previous myocardial infarction, n (%) | 4 (8) | 23 (23) |
| Previous PCI, n (%) | 3 (6) | 20 (20) |
| Previous stroke/TIA, n (%) | 4 (8) | 0 (0) |
| Hypertension, n (%) | 32 (65) | 75 (77) |
| Diabetes mellitus, n (%) | 12 (24) | 13 (13) |
| Habitual eGFR, mL/min/1.73 m^2^, median (IQR) | 72 (56–80) | 61 (52–71) |
| **Footnote.**  CCS controls were matched 2:1 to sepsis patients on age, gender and number of diseased epicardial vessels.  **Abbreviations.**  BMI = body mass index; CAD = coronary artery disease; CCS = chronic coronary syndrome; eGFR = estimated glomerular filtration rate; IQR = interquartile range; PCI = percutaneous coronary intervention; TIA = transient ischaemic attack. | | |

**Figure S1.** **Extended patient flow chart**

**Patients with sepsis or septic shock treated in ICU/IMCU and**

**prospectively screened for eligibility**

(*n* = 416)

June 1, 2019 – December 20, 2024

**Excluded before enrolment** (n = 354)

**Did not meet inclusion criteria:**

- hs-cTnT unavailable within 48 h (n = 15)
- hs-cTnT <15 ng/L (n = 27)
- Expected survival <1 year (n = 30)

**Unable to provide informed consent:**

- Cognitive impairment or dementia (n = 51)
- Severe psychiatric illness (n = 9)
- Acute delirium (n = 18)

**Clinical deterioration before inclusion:**

- Rapid in-hospital death before inclusion (n = 24)

**Pre-specified exclusion criteria:**

- Previous coronary artery bypass grafting (n = 14)
- Pre-admission left ventricular ejection fraction ≤39% (n = 28)
- Chronic kidney disease (eGFR < 30 mL/min/1.73 m²) (n = 48)
- Asthma (n = 24)
- Infective endocarditis (n = 5)

**Additional safety and feasibility exclusions:**

- Clinical instability beyond day 8 (n = 18)
- Mechanical aortic valve or prior transcatheter valve implantation (n = 4)
- Severe mitral valve disease (n = 3)
- Unresolved acute kidney failure by day 10 (n = 5)
- Transferred to another hospital before inclusion (n = 4)
- Sepsis requiring surgery (n = 16)
- Acute haemorrhage (n = 6)

**Declined participation:**

- Patient refused study inclusion (n = 5)

**Enrolled patients** (n = 62)

**Did not undergo coronary angiography** (n = 7)

- Declined angiography after enrolment (n = 3)
- Angiography not recommended after enrolment (n = 4)**^1^**

**Coronary angiography cohort** (n = 55)

**Coronary angiography without coronary microvascular assessment** (n = 6)

- Severe LAD stenosis / vasospasm / unfavourable anatomy / unable to remain supine (n = 4)
- Delayed angiography >10 days (n = 2)**^2^**

**Coronary angiography with coronary microvascular assessment** (n = 49)**^3^**

**^1^** Angiography not recommended post-enrolment: acute-on-chronic kidney failure; previously unreported contrast allergy; gastrointestinal bleeding; acute severe thrombocytopenia.

**^2^** Severe progressive soft-tissue infection (n = 1); recurrent clinical instability post-enrolment (n = 1).

**^3^** Complete coronary microvascular physiological measurements were obtained in 49 patients, who comprised the complete coronary physiology cohort used for analyses involving IMR, MRR.

**Abbreviations:** eGFR = estimated glomerular filtration rate; hs-cTnT = high-sensitivity cardiac troponin T; ICU = intensive care unit; IMCU = intermediate care unit; IMR = index of microcirculatory resistance; LAD = left anterior descending artery; MRR = microvascular resistance reserve.

**Per protocol** (*n*=49)^4^

**Figure S2. Distribution of cardiac biomarkers**

**(A)** Histogram of the highest high-sensitivity cardiac troponin T (hs-cTnT) concentration measured within 48 hours of sepsis onset (displayed range 15–1000 ng/L; n = 55). Four patients with hs-cTnT concentrations >1000 ng/L (maximum 3360 ng/L) are not shown.
**(B)** Histogram of the highest N-terminal pro-B-type natriuretic peptide (NT-proBNP) concentration measured within 48 hours of sepsis onset (displayed range 100–35,000 ng/L; n = 55).


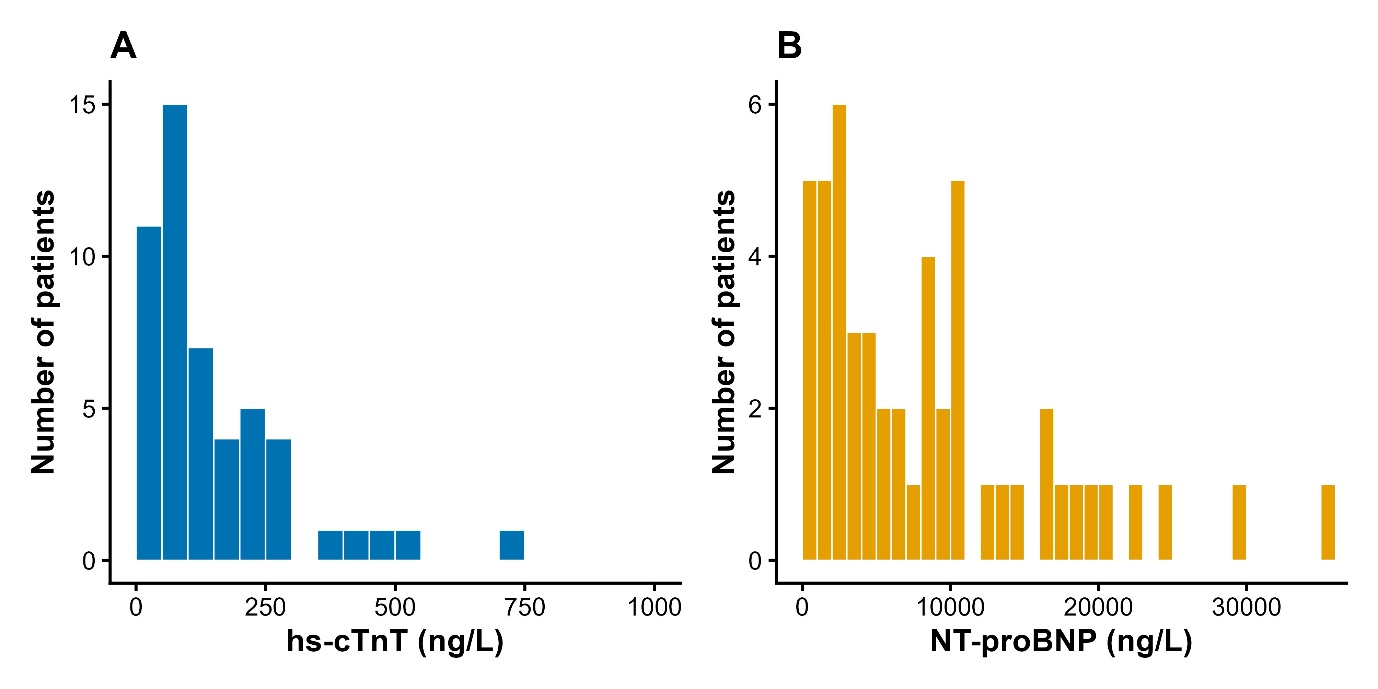


**Figure S3. Associations between cardiac biomarkers and echocardiographic indices in sepsis.** Standardised β-coefficients (95% CI) from linear regression analyses showing associations between hs-cTnT and NT-proBNP and selected indices of left and right ventricular function and ventriculo-arterial and right ventricular-pulmonary arterial coupling. Red symbols indicate statistically significant associations (*P* < 0.050), whereas grey symbols indicate non-significant associations.

Abbreviations: E/A ratio = ratio of early (E) to late (A) diastolic transmitral filling velocities; LV = left ventricle; LVEF = left ventricular ejection fraction; LV-LWFS = left ventricular longitudinal wall fractional shortening; RV = right ventricle; SPAP = systolic pulmonary arterial pressure; TAPSE = tricuspid annular plane systolic excursion; VAC = ventriculo-arterial coupling.


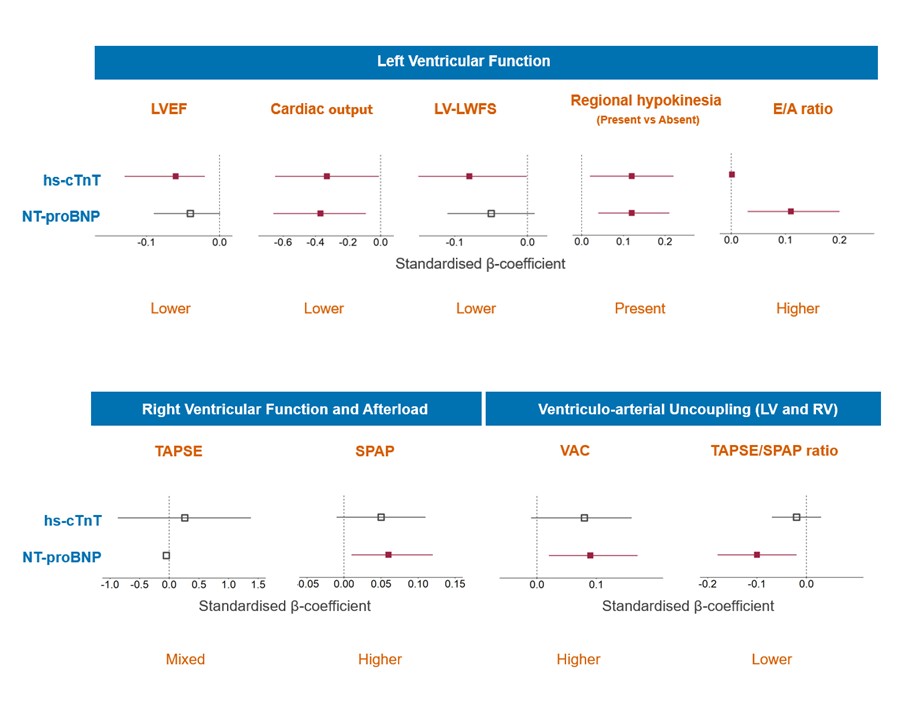


**Supplementary References**

1. Lang RM, Badano LP, Mor-Avi V, Afilalo J, Armstrong A, Ernande L, et al. Recommendations for cardiac chamber quantification by echocardiography in adults: an update from the American Society of Echocardiography and the European Association of Cardiovascular Imaging. J Am Soc Echocardiogr. 2015;28(1):1-39.e14.

2. Huang SJ, Ting I, Huang AM, Slama M, McLean AS. Longitudinal wall fractional shortening: an M-mode index based on mitral annular plane systolic excursion (MAPSE) that correlates and predicts left ventricular longitudinal strain (LVLS) in intensive care patients. Crit Care. 2017;21(1):292.

3. Orde S, Huang SJ, McLean AS. Speckle tracking echocardiography in the critically ill: enticing research with minimal clinical practicality or the answer to non-invasive cardiac assessment? Anaesth Intensive Care. 2016;44(5):542-51.

4. Blixt PJ, Nguyen M, Cholley B, Hammarskjöld F, Toiron A, Bouhemad B, et al. Association between left ventricular systolic function parameters and myocardial injury, organ failure and mortality in patients with septic shock. Ann Intensive Care. 2024;14(1):12.

5. Johansson Blixt P, Chew MS, Åhman R, de Geer L, Blomqwist L, Åström Aneq M, et al. Left ventricular longitudinal wall fractional shortening accurately predicts longitudinal strain in critically ill patients with septic shock. Ann Intensive Care. 2021;11(1):52.

6. Chen CH, Fetics B, Nevo E, Rochitte CE, Chiou KR, Ding PA, et al. Noninvasive single-beat determination of left ventricular end-systolic elastance in humans. J Am Coll Cardiol. 2001;38(7):2028-34.

7. Saeed S, Holm H, Nilsson PM. Ventricular-arterial coupling: definition, pathophysiology and therapeutic targets in cardiovascular disease. Expert Rev Cardiovasc Ther. 2021;19(8):753-61.

8. Robinson S, Ring L, Oxborough D, Harkness A, Bennett S, Rana B, et al. The assessment of left ventricular diastolic function: guidance and recommendations from the British Society of Echocardiography. Echo Res Pract. 2024;11(1):16.

9. Rudski LG, Lai WW, Afilalo J, Hua L, Handschumacher MD, Chandrasekaran K, et al. Guidelines for the echocardiographic assessment of the right heart in adults: a report from the American Society of Echocardiography endorsed by the European Association of Echocardiography, a registered branch of the European Society of Cardiology, and the Canadian Society of Echocardiography. J Am Soc Echocardiogr. 2010;23(7):685-713; quiz 86-8.

10. Nasser MF, Jabri A, Limaye S, Sharma S, Hamade H, Mhanna M, et al. Echocardiographic Evaluation of Pulmonary Embolism: A Review. J Am Soc Echocardiogr. 2023;36(9):906-12.

11. Yamazaki T, Saito Y, Yamashita D, Kitahara H, Kobayashi Y. Factors Associated with Impaired Resistive Reserve Ratio and Microvascular Resistance Reserve. Diagnostics (Basel). 2023;13(5).

12. Layland J, Carrick D, McEntegart M, Ahmed N, Payne A, McClure J, et al. Vasodilatory capacity of the coronary microcirculation is preserved in selected patients with non-ST-segment-elevation myocardial infarction. Circ Cardiovasc Interv. 2013;6(3):231-6.

13. Yong AS, Layland J, Fearon WF, Ho M, Shah MG, Daniels D, et al. Calculation of the index of microcirculatory resistance without coronary wedge pressure measurement in the presence of epicardial stenosis. JACC Cardiovasc Interv. 2013;6(1):53-8.

14. Pijls NH, De Bruyne B, Peels K, Van Der Voort PH, Bonnier HJ, Bartunek JKJJ, et al. Measurement of fractional flow reserve to assess the functional severity of coronary-artery stenoses. N Engl J Med. 1996;334(26):1703-8.

15. De Bruyne B, Pijls NHJ, Gallinoro E, Candreva A, Fournier S, Keulards DCJ, et al. Microvascular Resistance Reserve for Assessment of Coronary Microvascular Function: JACC Technology Corner. J Am Coll Cardiol. 2021;78(15):1541-9.
